# An *ALPK3* truncation variant causing autosomal dominant hypertrophic cardiomyopathy is partially rescued by mavacamten

**DOI:** 10.1038/s41598-025-94371-w

**Published:** 2025-03-24

**Authors:** Lisa Leinhos, Paul Robinson, Giulia Poloni, Sophie Broadway-Stringer, Julia Beglov, Adam B. Lokman, Gillian Douglas, Sajjad Nuthay, Oveena Fonseka, Manuel Schmid, Evie Singer, Charlotte Hooper, Kate Thomson, Richard D. Bagnall, Jodie Ingles, Christopher Semsarian, Elizabeth Ormondroyd, Christopher N. Toepfer, Benjamin Davies, Charles Redwood, Hugh Watkins, Katja Gehmlich

**Affiliations:** 1 Division of Cardiovascular Medicine, Radcliffe Department of Medicine and British Heart Foundation Centre of Research Excellence Oxford, University of Oxford, Oxford, UK; 2 Department of Cardiovascular Sciences, School of Medical Sciences, College of Medicine and Health, Institute of Biomedical Research (IBR) room 229, University of Birmingham, Edgbaston, Birmingham, B15 2TT UK; 3 Institute of Developmental and Regenerative Medicine, University of Oxford, Oxford, UK; 4 Agnes Ginges Centre for Molecular Cardiology at Centenary Institute, The University of Sydney, Sydney, Australia; 5 Faculty of Medicine and Heath, The University of Sydney, Sydney, Australia; 6 Genomics and Inherited Disease Program, Garvan Institute of Medical Research and University of New South Wales, Sydney, Australia; 7 Department of Cardiology, Royal Prince Alfred Hospital, Sydney, NSW Australia; 8 NIHR Oxford Biomedical Research Centre, Oxford, UK; 9 Wellcome Centre for Human Genetics, Transgenic Core, University of Oxford, Oxford, UK

**Keywords:** ALPK3, Hypertrophic cardiomyopathy, Genetic cardiac disease modelling, Mouse model, Mavacamten, Myofilament, Cardiovascular biology, Cell biology, Genetics, Molecular biology, Physiology

## Abstract

The *ALPK3* gene encodes alpha-protein kinase 3, a cardiac pseudo-kinase of unknown function. Heterozygous truncating variants (*ALPK3tv)* can cause dominant adult-onset hypertrophic cardiomyopathy (HCM). Here we confirm an excess of *ALPK3tv* in sarcomere-gene negative HCM patients. Moreover, we generated a novel knock-in mouse model carrying an *ALPK3tv* (K201X). Homozygous animals displayed hypertrophy and systolic dysfunction. Heterozygous animals demonstrated no obvious baseline; however, they had an aggravated hypertrophic response upon chronic adrenergic challenge. Isolated, unloaded cardiomyocytes from heterozygous and homozygous mice showed reduced basal sarcomere length with prolonged relaxation, whilst calcium transients showed increased diastolic calcium levels. Protein kinase A-mediated phosphorylation, including that of cardiac troponin I, was significantly decreased. In agreement with the cellular HCM phenotype, reduced ratios of myosin heads in the super-relaxed state were measured. Contractile and calcium handling defects were partly corrected by treatment with mavacamten, a novel myosin inhibitor. For the first time with a non-sarcomere HCM variant, we have demonstrated hallmark changes in cardiac contractility and calcium handling. Mavacamten is able to partially rescue the cellular phenotype, hence could be beneficial to HCM patients with *ALPK3tv.* Moreover, our data points at a potential role of ALPK3 as a modulator of protein kinase A signalling.

## Introduction

Alpha-protein kinase 3 (ALPK3) was initially classified as an atypical protein kinase, with cardiac and skeletal muscle specific expression. The protein contains two amino-terminal immunoglobulin-like domains (Ig) and a carboxy-terminal, putative kinase domain. Based on sequence homology, it belongs to the unique family of eukaryotic alpha-protein kinases^1^. Two studies have presented strong evidence that the protein is a pseudokinase without kinase activity^2,3^; although another study has shown that the presence of ALPK3 regulates the phosphorylation of certain myofilament proteins; this is likely to be an indirect effect^4^.

Early studies reported that nuclear ALPK3 plays a major role in cardiac differentiation and cardiomyocyte proliferation^5^. More recently, the protein has been shown to localise to the M-band in mature cardiomyocytes and additionally to the nuclear envelope in differentiating induced pluripotent stem cell (iPSC) derived cardiomyocytes. In both structures, it may act as a scaffold for M-band proteins such as myomesin, and buffer force^2^. It was also demonstrated that ALPK3 interacts with p62 within the M-band, suggesting a role in maintaining sarcomeric proteostasis^4^.

Genetic variants in the *ALPK3* gene have been associated with cardiomyopathy^6–10^. Recessive (homozygous) *ALPK3tv* cause an early-onset severe form of cardiomyopathy *in utero* or in early infancy, which is characterised by systolic dysfunction, dilatation and hypertrophy^6,11,12^. Some reports also described extra-cardiac features, e.g. musculoskeletal and dysmorphic abnormalities, in the presence of recessive *ALPK3tv*. The majority of heterozygous *ALPK3tv* carriers in these families did not display an overt cardiomyopathy phenotype, even in adulthood. Nevertheless, 2 out of 10 (20%) heterozygous family members presented with adult onset hypertrophic cardiomyopathy (HCM)^6^.

More recently, data from large cohort studies have supported the hypothesis that heterozygous *ALPK3tv* are causal of autosomal dominant HCM. Lopes et al. detected heterozygous *ALPK3tv* in ~ 1.6% of HCM cases, and found evidence of enrichment in cases compared to the gnomAD population (odds ratio [OR] ~ 16)^9^. Evidence of enrichment of rare heterozygous *ALPK3tv* has since been described in other HCM cohorts^8,13^. Additionally, co-segregation of a heterozygous *ALPK3tv* with autosomal dominant HCM was described in a four-generation family with seven affected members^8^.

In this study, we undertook case control analyses to confirm the level of enrichment of rare *ALPK3tv* in our cohort of HCM patients. To examine the functional consequences, we generated a novel knock-in mouse model carrying an *ALPK3tv* (K201X) found in an adult onset autosomal dominant HCM patient. Homozygous mice mimicked features seen in patients with recessive *ALPK3tv*, namely reduced systolic function, dilatation and hypertrophy, while the heterozygous mice showed no overt phenotype in vivo.

Pathological dysfunction that shared features seen with sarcomere-related HCM was evident in isolated cardiomyocytes, both in the heterozygous and homozygous setting. We also demonstrated that mavacamten, a drug licenced to treat patients with obstructive HCM, reverted some aspects of the cellular dysfunction observed in cardiomyocytes of our mouse model.

## Materials and methods

### Ethical statement: patient data

All research involving humans has been performed in accordance with the 1964 Declaration of Helsinki and its amendments. All patients provided informed consent for the study (i.e. genomic sequencing, and analysis of demographic, clinical, and family history data). The study is part of the NIHR Bioresource Rare Disease HCM project (BRRD), which was approved by the ‘East of England - Cambridge South Research Ethics Committee’ (reference 13/EE/0325). All research was performed in accordance with relevant guidelines/regulations.

### Patient case-control analysis

Rare variant burden analyses were performed as described previously^14^, using rare variant data available from 230 unrelated, sarcomere variant negative, HCM cases and 6,219 unrelated individuals, recruited to other rare disease projects within the BRRD. Well-established sarcomere-related HCM genes (*MYBPC3*,* MYH7*,* TNNI3*,* TNNT2*,* MYL2*,* MYL3*,* ACTC1*,* TPM1*), genes for common differential diagnoses (*PRKAG2*,* GLA*,* FHL1*), and other more rarely associated, but validated, HCM genes (*CSRP3*,* PLN*) were included in the genetic testing.

The proportion of rare (total minor allelic frequency < 1 × 10^− 4^ in gnomADvs2.1.1) missense, truncating (frameshift, nonsense, splice donor/acceptors), non-truncating (missense, in-frame insertions and deletions) and synonymous *ALPK3* variants was compared between HCM cases and controls. Fisher’s exact test (FET) and odds ratios (OR) with 95% confidence intervals (CI) were calculated.

Variants were annotated according to the MANE transcripts (NM_929778.5; ENST0000025888.6). Variants were reviewed and classified according to clinical variant interpretation guidelines^15^.

### Ethical statement: animal procedures

Experimental animal studies were performed in accordance with the relevant guidelines and regulation, which are stipulated by the UK Home Office guidelines. The study was approved by institutional ethical review board (‘Animal Welfare and Ethical Review Body’ of the University of Oxford, reference Project Licence PPL PDCE16CB0). All experiments were performed in accordance with ARRIVE guidelines.

### Generation of mouse model

A novel knock-in (KI) mouse model carrying one of the *ALPK3tv* found in a patient with autosomal dominant HCM (*ALPK3* p. K201X), was generated in house by the Genome Engineering Core Facility at the Wellcome Centre for Human Genetics.

To incorporate the desired c.601A > T variant in a mouse model (and two further silent changes, Fig. S1A), a CRISPR/Cas9 nuclease was designed against murine *Alpk3* exon 5 (ENSMUSE00000506542) the CRISPOR design tool (crispor.telfor.net) along with a silent mutation to remove the PAM sequence adjacent to the target sequence. For easy detection of the recombinant allele, an additional silent mutation was introduced, resolving in a unique HindIII restriction site. One target site was identified close to the Lysine residue according to genome-wide specificity (lack of many significant off-target sites). A sgRNA designed against the selected guide sequence (5’- GCATGGAAAAGAGGCTTCAG-3’) was purchased from Synthego. A founder mouse was generated by microinjecting the 139 nt ssODN and the guide-RNA for the designed CRISPR/Cas9 nuclease into fertilized C57BL/6J zygotes.

Animals were backcrossed onto C57BL/6JOlaHsd (Envigo RMS (UK) Ltd) for at least 6 generations until congenic before phenotyping. Consistent genetic background of mice was confirmed using MiniMUGA genotyping array analysis via Transnetyx^®^ service^16^.

Genotyping was performed using the REDExtract-N-Amp™ Tissue PCR Kit (Sigma) and primer pair Alpk3-F1 5’- AGAAGATGCTGCCATCTACCAA-3’ and Alpk3-R1 5’- TGACCTCGCAGATGTATGTCAG-3’. The 470 bp amplicon was digested with *HindIII* restriction enzyme (NEB) and analysed by agarose electrophoresis (1.5% agarose in Tris-Borate-EDTA buffer, Fisher). The wildtype allele was not digested (470 bp fragment), while the *Alpk3* K201X allele was digested into two fragments of 183 bp and 287 bp.

### Animal husbandry

Animals were maintained in standardised pathogen-free ventilated cages with food and water supply *ad libitum* and a 13 h light / 11 h dark circadian rhythm (150–200 lx cool white LED light, measured at the cage floor), with the only reported positives on health screening over the entire time course of these studies being for. *Chilomastix Sp* and *Entamoeba muris*. Where possible, animals were housed in social groups of mixed genotypes, except for animals under adrenergic challenge studies, which were single-housed with additional enrichment for refinement reasons.

All studies were carried out using adult, male mice littermates, while females were used for breeding. Mice were housed in mixed genotype cages. Treatment groups were assigned at random and all data acquisition and analysis was performed blinded. Animals were culled by a schedule 1 method (i.e. cervical dislocation followed by confirmation of death via cessation of circulation); no drugs or chemicals were used for euthanasia.

### Echocardiography

Ultrasound in vivo phenotyping of the heart was performed on male mice under general anaesthesia using the Vevo 3100 micro-ultrasound system (FUJIFILM VisualSonics). Anaesthesia was induced by an induction chamber filled with 4% isoflurane in 2 L/min 100% oxygen and later maintained with 1.5 − 2% isoflurane (Zoetis) in 1 L/min 100% oxygen delivered through a fitted nose-cone. The animal’s body temperature was maintained at 37 ºC using a heated platform during the procedure and vital functions were monitored via an embedded electrode system. Cardiac structure and function were measured at comparable physiologically relevant heart rates (where possible between 460 and 480 bpm) for 30 to max. 60 min, according to the approved PPL guidelines. Parameters were analysed and calculated using the Vevo LAB software (version 5.6.1).

### Adrenergic challenge via osmotic minipumps

To induce hypertrophic pathways and to explore consequences on cardiac performance, chronic adrenergic challenge with isoprenaline/phenylephrine (Iso/PE) delivered by osmotic minipumps was performed. Male animals were assigned to treatment groups at random and underwent pre-implant baseline echocardiography.

**Fig. 1:**
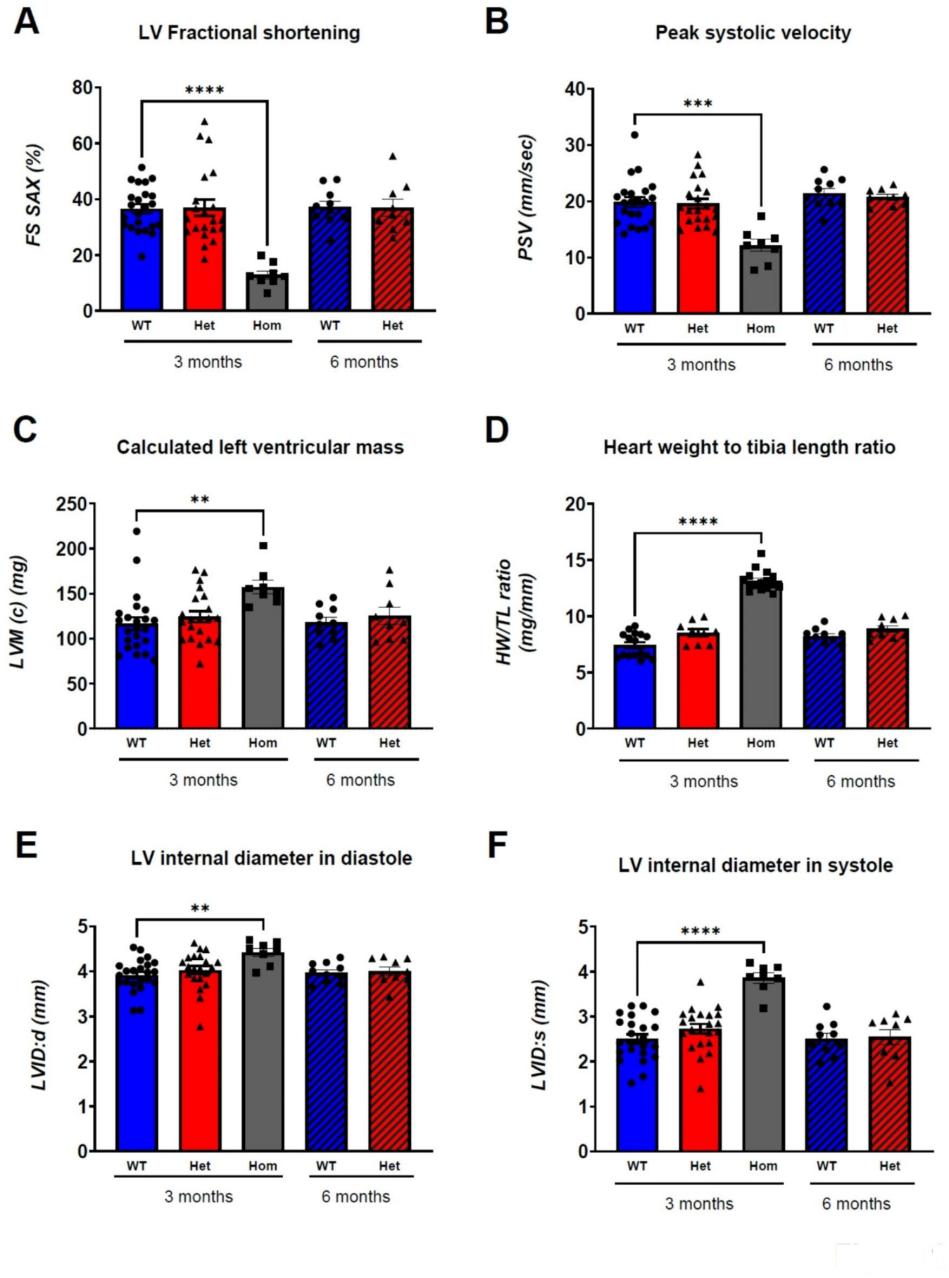
Phenotype of the novel *Alpk3 K201X* mouse model. Cardiac structure and function were assessed via echocardiography in wildtype (WT), heterozygous (Het) and homozygous (Hom) *Alpk3* K201X animals at 3 months. WT and Het animals were also assessed at 6 months. Fractional shortening using the short axis view (FS SAX, **A**), peak systolic velocity (PSV, **B**), calculated left ventricular mass (LVM (c), **C**), heart weight (HW) to tibia length (TL) ratio (**D**), and left ventricular internal diameter in diastole (LVID: d, **E**) and systole (LVID: s, **F**) are shown. Values are presented as mean ± SEM. Kruskal-Wallis test indicates significant changes in Hom mice; **p < 0.01, ****p < 0.0001 versus WT 3 months. To compare WT and Het mice at 6 months, unpaired Student’s t-test was performed and no significant changes were observed. For a full set of echocardiographic parameters and n numbers per parameter see (Table S3).

Baseline echocardiography data were also used as additional values for the for the 3-month-old WT and Hom *Alpk3* K201X comparison (*n* = 23 WT, *n* = 8 Hom, Fig. 1, Table S3) and for the 3-month-old WT and Het *Alpk3* K201X comparison (*n* = 23 WT, *n* = 21 Het, *n* = 18 Hom, Fig. S2, Table S4).

All animals received an injection of buprenorphine (0.05 mg/kg body weight of 0.3 mg/mLVetergesic^®^ Ceva) subcutaneously for pain prevention before the surgical procedure. Osmotic minipumps (ALZET 1002) for drug administration were implanted subcutaneously in a sterile midscapular incision procedure under general anaesthesia (constant supply of 2% isoflurane in 1 L/min 100% oxygen, as described above).

Treatment groups received isoprenaline and phenylephrine (Iso/PE; in hydrochloride forms, Sigma Aldrich), which are β- and α-adrenergic agonists, at a concentration of 15 mg kg^− 1^ body weight each in 0.9% NaCl (Vetivex ^®^ 1, Dechra) at a constant flow rate of 0.25 µl/hr per day for 14 days in total. Control groups received 0.9% NaCl as a placebo via osmotic minipumps at the same flow rate and for the same amount of time. All animals were monitored daily according to the PPL guidelines, two animals were excluded from the study following animal welfare guidelines. Echocardiography was performed 13 days post-surgery, followed by scarification and organ harvest on day 14 for further ex vivo analysis.

### Ex vivo measurements

Mice were sacrificed by cervical dislocation; the total body weight was recorded. Hearts were dissected using sterile surgical tools. Whole hearts were washed in PBS and weighed. Ventricular tissue was snap frozen in liquid nitrogen and stored at − 80 °C for future analysis.

To determine the tibia length, whole legs were dissected and digested in 0.8 M KOH overnight. Following the digestion, the tibia was measured and heart weight to tibia length ratio calculated.

### Contractility and Ca^2+^ measurements

Mouse left ventricular cardiomyocyte isolation was performed by Langendorff perfusion with Tyrode’s buffer (130 mM NaCl, 5.6 mM KCl, 3.5mM MgCl_2_), 5 mM 4-(2-hydroxyethyl)-1- piperazineethanesulphonic acid, HEPES, 0.4 mM Na_2_HPO_4_) containing 27 µg/ml liberase as previously described^17^. Digestion of extracellular matrix by protease was quenched by diluting the resultant cell suspension with 3 volumes of 1% bovine serum albumin (BSA) in Tyrode’s solution and made sequentially Ca^2+^ competent by settling cells under gravity to form a loose pellet, removing the supernatant solution and adding back Tyrode’s with 1% BSA and 500 µM CaCl_2_, then 1% BSA containing 1 mM CaCl_2_ and finally resuspended in storage buffer (120 mM NaCl, 5.6 mM KCl, 5 mM MgSO_4_, 5 mM sodium pyruvate, 10 mM HEPES, 200 mM glucose, 200 mM taurine, 0.5 mM CaCl_2_) for further use.

Cell suspensions were divided, 10% was used for immunofluorescence slide preparation, 25% used for western blot sample preparation, 15% reserved for unloaded shortening measurements (without Ca^2+^ indicator loading) and the remaining 50% were incubated with fura2-AM-ester (fura2) for Ca^2+^ transient and pairwise unloaded sarcomere shortening measurements using IonOptix µstep apparatus. Cardiomyocyte contraction was assessed by fast Fourier transform of sarcomeric striations using phase-contrast microscopy. Ratiometric measurement of intracellular calcium ([Ca^2+^]_i_) transients were measured using dual excitation at 365 and 380 nm and measured emission at 520 nm by photomultiplier light acquisition.

Cardiomyocytes were loaded with fura2 (2 µM) in black Eppendorf tubes for 5 min in Tyrode’s solution containing 250 µM CaCl_2_ + Pluronic F127, cells were washed for 10 min in Tyrode’s containing 500 µM CaCl_2_ to remove any unloaded fura2 dye. At this point fura2 loaded cells were split with 50% of the cell suspension treated with 0.5 µM mavacamten and the remaining 50% treated with a DMSO vehicle control. Loaded cardiomyocytes were then placed in a glass-bottom chamber, mounted onto an inverted microscope, and perfused at 35 ± 1 °C with 1.4 mM CaCl_2_ Tyrode’s solution containing 0.5 µM mavacamten or DMSO depending on prior treatment of the cells. Cardiomyocytes were field-stimulated at 40 volts and a frequency of 3 Hz for several minutes to allow steady-state [Ca^2+^]_i_ transients to be reached before recordings were taken. For each set of recordings, five background fluorescence measurements were taken from a cell-free field. [Ca^2+^]_i_ transients were measured as a function of the ratio of emissions from 365/380 excitation wavelength after subtraction of the background fluorescence (IonOptix) relative fluorescence was calibrated to [Ca^2+^] using the single exponential equation

$$\:\left[{Ca}^{2+}\right]=-0.9853+2.5556\times\:{F365/380}^{3.3455}$$

using the Ca^2+^ ionophore permeabilization method previously described^18,19^. Average contractility and Ca^2+^ transients were determined from at least 10 individual raw tracings. Cells with poor morphology (e.g., excessive blebbing or asynchronous contraction) were not measured. Parameters were extracted using IonWizard 6.6.

### Western blotting

Western blots were performed using 4–15% mini-protean TGX gels (BioRad). For Phospho-Ser23/24 cTnI blots, a standard wet transfer on to PVDF membrane was used with subsequent blocking with 5% skimmed milk powder in Tris buffered saline 0.1% Tween-20. Membranes were probed with a 1:2,000 dilution of anti Phospho-Ser23/24 TnI antibody (Cell Signalling) and 1:6,000 diluting of anti-rabbit-HRP secondary (Promega). Loading controls following antibody stripping with Restore Plus reagent (Thermo Fisher Scientific) used 1:5000 anti-cTnI (Abcam) with 1:15,000 anti-mouse-HRP (Promega) secondary and finally a 1:10,000 anti-GAPDH (Abcam) with a 1:30,000 anti-rabbit-HRP secondary. To measure RRXS/T protein kinase A target levels, a semi dry transfer was preformed using iBlot2 PVDF cassettes (Thermo Fisher Scientific), blocking and antibody incubation was performed in 5% BSA-TBST solution. A 1:1,000 dilution of anti-phospho-RRXS/T (Cell Signalling^20^) primary and 1:3,000 anti-rabbit-HRP secondary was used to probe for target proteins. An equal loading of all samples was run in parallel on a separate gel and stained with 5% Coomassie brilliant blue (Merck) to assess total protein loading. Densitometric measurements for all blots was performed using Image Lab following development of blot membranes with Dura ECL reagent (Thermo) using a GelDoc imaging system installed with software version 5.1 (BioRad). For exposure, the setting is “Chemi-High Sensitivity” (with 4 × 4 binning) and “Image Exposure: The software will automatically optimise the exposure for Intense Bands” options were used. For Phospho-RRXS/T blots the entire lane was analysed using a band detection threshold of 95% and a cumulative densitometry was calculated. We excluded the over-exposed cTnI band at 24 kDa by manual removal. Coomassie stained loading controls showed a consistent actin band at 42 kDa which was analysed by densitometry as a loading control.

### Statistics

In vivo data was analysed blind (to genotype and/or treatment groups). All values are given as mean ± standard error of mean (SEM). Data was tested for normality using the Kolmogorov-Smirnov test. To compare two unpaired sample groups, normally distributed data were analysed by Student’s t-test, and data that was not normally distributed were analysed by Mann-Whitney U-test. For comparison of three groups with normally distributed data, 1-way ANOVA followed by Tukey’s post-hoc test was used. For non-normally distributed data, Kruskal-Wallis test with Dunn’s test for multiple comparisons was used. Chronic adrenergic challenge response was analysed by 2-way ANOVA (with Tukey post-hoc test for multiple comparisons).

For contractility and calcium transient measurements, all extracted parameters for each genotype and treatment group were split by individual mouse, tested for normality using D’Agostino & Pearson test and analysed for statistical significance for hierarchical cluster statistical analysis using nested 1-way ANOVA as previously described^21^. All values are plotted as median ± 95% confidence interval due to non-normal data distribution and discussed and tabulated as mean ± SEM because nested statistical analysis is agnostic to data distribution.

All statistical analyses were performed blinded to genotype and treatment (where applicable) with GraphPad Prism 9.4.1.

Annotations used: * *p* < 0.05, ** *p* < 0.01, *** *p* < 0.001, **** *p* < 0.0001 versus WT, otherwise considered not significant (*p* > 0.05); n indicates number of animals in each group.

## Results

### Heterozygous *ALPK3tv* are enriched in patients with autosomal dominant adult onset HCM

We interrogated *ALPK3* genetic variants in a cohort of 230 HCM patients, in whom genetic screening had not identified a pathogenic genetic variant in sarcomere genes, and compared them to 6,219 controls without HCM. *ALPK3tv* were detected in 5 of 230 HCM cases (2.2%) and 9 of 6,219 controls (0.14%) (Fig. S1A, Table S1). This was a statistically significant excess of rare *ALPK3tv* in HCM cases compared to controls (OR 16.07, 95% CI 4.27–52.05; FET *p* = 0.0001), (Table S2). In contrast, there was no significant difference in the proportion of rare missense, non-truncating, or synonymous variants in HCM cases compared to controls (Table S2). This supports findings from recent studies^8,9,13^ that *ALPK3tv* cause autosomal dominant adult onset HCM.

### A novel mouse model for *ALPK3tv*

One of the *ALPK3tv* from our HCM cohort (Fig. S1A, Table S1) was used to generate a mouse model for *ALPK3*-associated HCM, *Alpk3* K201X (Fig. S1B,C). We chose a human autosomal dominant *ALPK3tv*, as opposed to a full gene knockout^22^, to account for potential dominant negative effects of the truncated protein, which in case of truncation at Lysine 201 would lack the putative kinase domain. Since recessive (e.g. homozygous) *ALPK3tv* can lead to early onset cardiomyopathy in patients, the cardiac structure and function of homozygous (Hom) mice with the *Alpk3* K201X truncation variant were analysed and compared to wildtype (WT) animals alongside heterozygous (Het) animals using echocardiography (Fig. 1, S2, Table S3).

Homozygous *Alpk3* K201X animals were viable and fertile, and showed no gross extra-cardiac abnormalities. Their hearts displayed a significant impairment of systolic function compared to WT mice at 3 months, evidenced by a significant reduction of fractional shortening (Fig. 1A) and peak systolic velocity (Fig. 1B). Moreover, these mice displayed features of hypertrophy, e.g. increased left ventricular mass (Fig. 1C) on echocardiography. In support, homozygous mouse hearts were enlarged (Fig. S2A) and had increased heart weight to tibia length ratio on gravimetry (Fig. 1D), but wall thicknesses were preserved (Table S3). Ventricular dilatation was evidenced by increased internal diameter of the left ventricle in both diastole and systole (Fig. 1E, F). These results suggest that the newly generated mouse model recapitulates expected cardiac features of human recessive *ALPK3tv*, namely cardiac hypertrophy, dilatation and systolic dysfunction.

At molecular level, the *Alpk3* K201X variant undergoes partial nonsense-mediated decay, with ~ 72 and ~ 27% of *Alpk3* transcript level in heterozygous and homozygous *Alpk3* K201X hearts compared to WT (Fig. S1C). Moreover, homozygous hearts displayed induction of transcripts related to the foetal gene programme and hypertrophic signalling in support of cardiomyopathy in these hearts (Fig. S3).

A side-by-side comparison of our novel mouse with the previously published *Alpk3* knockout (KO) model^2^ showed identical phenotypes on echocardiography (Table S4) and qPCR (Fig. S3).

### Heterozygous mice have normal baseline cardiac structure and function

Mice heterozygous for *Alpk3* K201X demonstrated normal cardiac function with no signs of hypertrophy or dilatation at 3 months. The analysed hearts were indistinguishable from WT littermates (Fig. 1, S2, Table S3).

In agreement with the absence of a phenotype on echocardiography, mice heterozygous for *Alpk3* K201X showed no induction of the foetal gene programme or transcripts related to hypertrophy on qPCR (Fig. S3). Histology was unremarkable in both heterozygous and homozygous setting (Fig. S4A), and there was no molecular evidence of fibrosis (Fig. S4B). However, isolated cardiomyocytes from both heterozygous and homozygous *Alpk3* K201X mice showed an increase in cell length, while cell width was normal (Fig. S4C).

As a role for ALPK3 in M-band organisation was implicated previously^2^, we analysed localisation of the M-band protein myomesin using immunofluorescence staining on cardiac tissue and isolated cells, but failed to detect any differences between the genotypes (Fig. S5). Please note that there is currently no working antibody available to localise Alpk3.

As HCM often manifests in adulthood in patients, adult mice of 6 months of age were also investigated. Similarly, at this age animals showed normal cardiac structure and function on echocardiography (Fig. 1, Table S3). Moreover, heart weight relative to tibia length was normal on gravimetry (Table S3).

In summary, mice heterozygous for *Alpk3* K201X failed to show signs of cardiomyopathy.

### Chronic adrenergic challenge aggravates the hypertrophic response in heterozygous mice

**Fig. 2:**
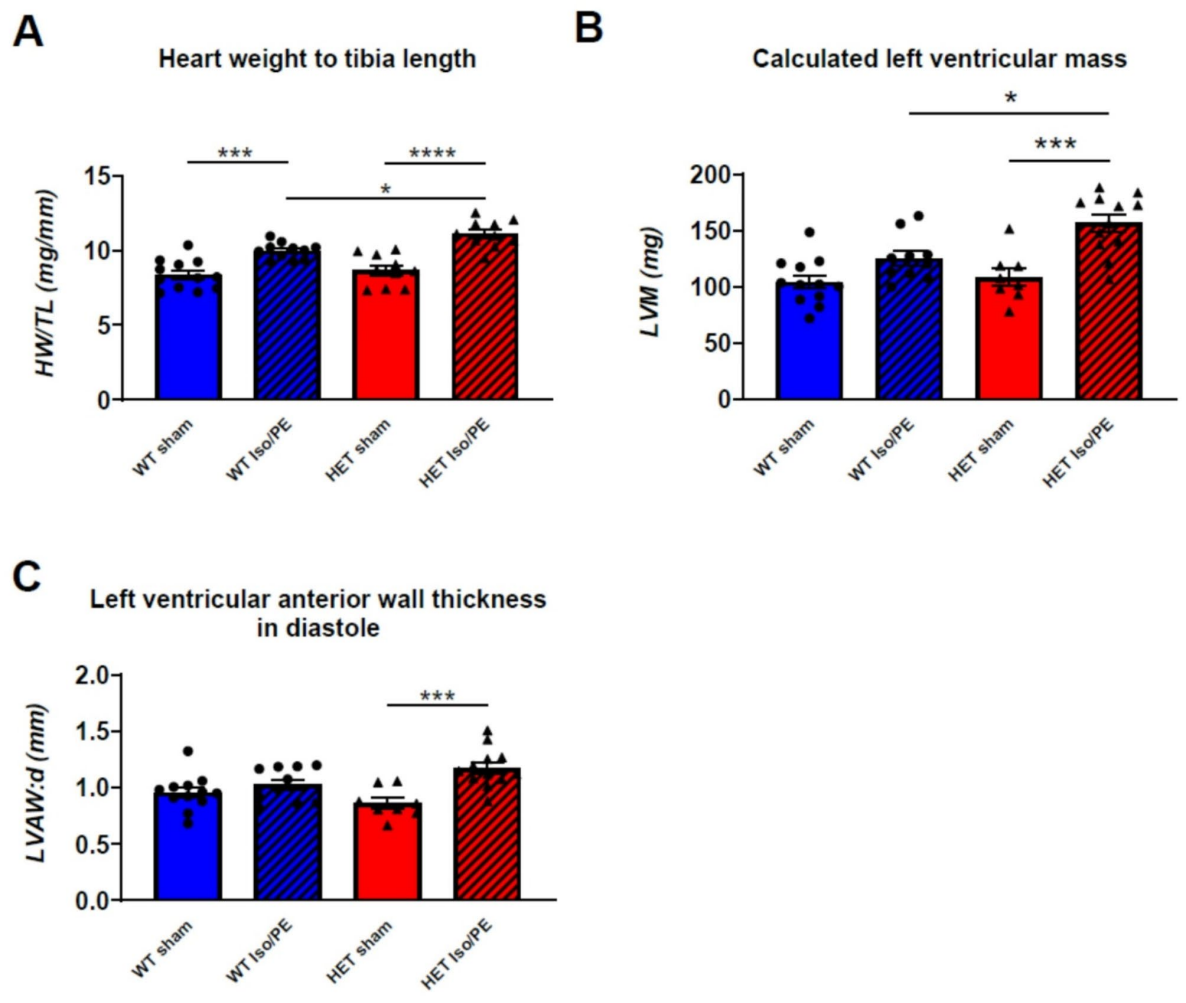
Chronic adrenergic challenge aggravates the hypertrophic response in heterozygous *Alpk3 K201X.* (**A**) The heart weight (HW) to tibia length (TL) ratio increased in both wildtype (WT) and heterozygous (Het) animals upon isoprenaline and phenylephrine (Iso/PE) treatment compared to their control groups (sham) treated with saline, indicating cardiac hypertrophy. There was a stronger response in Iso/PE treated Het compared to WT animals. WT sham *n* = 12; WT Iso/PE *n* = 11; Het sham *n* = 10; Het Iso/PE *n* = 10. (**B**) Het *Alpk3* K201X mice displayed an aggravated hypertrophic response upon chronic adrenergic challenge as evidenced by calculated left ventricular mass (LVM). (**C**) Left ventricular anterior wall thickness in diastole (LVAW: d) increased in challenged Het animals only. WT sham *n* = 12; WT Iso/PE *n* = 10; Het sham *n* = 8; Het Iso/PE *n* = 12 (for B and C). Values are presented as mean ± SEM. *p < 0.05, **p < 0.01, ****p < 0.0001 (2-way-ANOVA), otherwise considered not significant. For a full set of echocardiographic parameters please refer to (Table S5).

Since heterozygous *Alpk3tv* mice did not display an overt phenotype, we subjected animals to a chronic adrenergic challenge to induce a hypertrophic response via isoprenaline and phenylephrine (Iso/PE) treatment. The hypertrophic response of the murine hearts was examined upon two weeks of adrenergic stimulation using echocardiography. The heart weight to tibia length ratio increased in both WT and heterozygous animals upon drug-treatment compared to their control groups, confirming the treatment induced cardiac hypertrophy (Fig. 2A). There was a larger response in heterozygous *Alpk3* K201X hearts compared to WT hearts. In agreement, the calculated left ventricular mass based on echocardiography was enlarged in drug-treated heterozygous animals compared to corresponding control group and drug-treated WT animals (Fig. 2B). Left ventricular anterior wall thickness was increased in drug-treated heterozygous animals compared to control animals of the same genotype (Fig. 2C). However, systolic function was unchanged in both genotypes upon drug-treatment (ejection fraction and fractional shortening in Tab. S5). This experiment indicates that the mice carrying the heterozygous *Alpk3tv* have an aggravated hypertrophic response to chronic adrenergic challenge, while their systolic function is preserved.

### Cardiomyocytes from *Alpk3* K201X mice display reduced resting sarcomere length with prolonged relaxation

Sarcomeric HCM is associated with altered cardiomyocyte contractility and calcium transients. This is well documented for mouse models of HCM, even when subtle or no overt cardiac phenotypes are observed in vivo^23,24^. In order to investigate a potential cellular phenotype in our model, we isolated adult left ventricular cardiomyocytes from heterozygous and homozygous mice and compared their unloaded contractile parameters to cells isolated from WT mice.

**Fig. 3:**
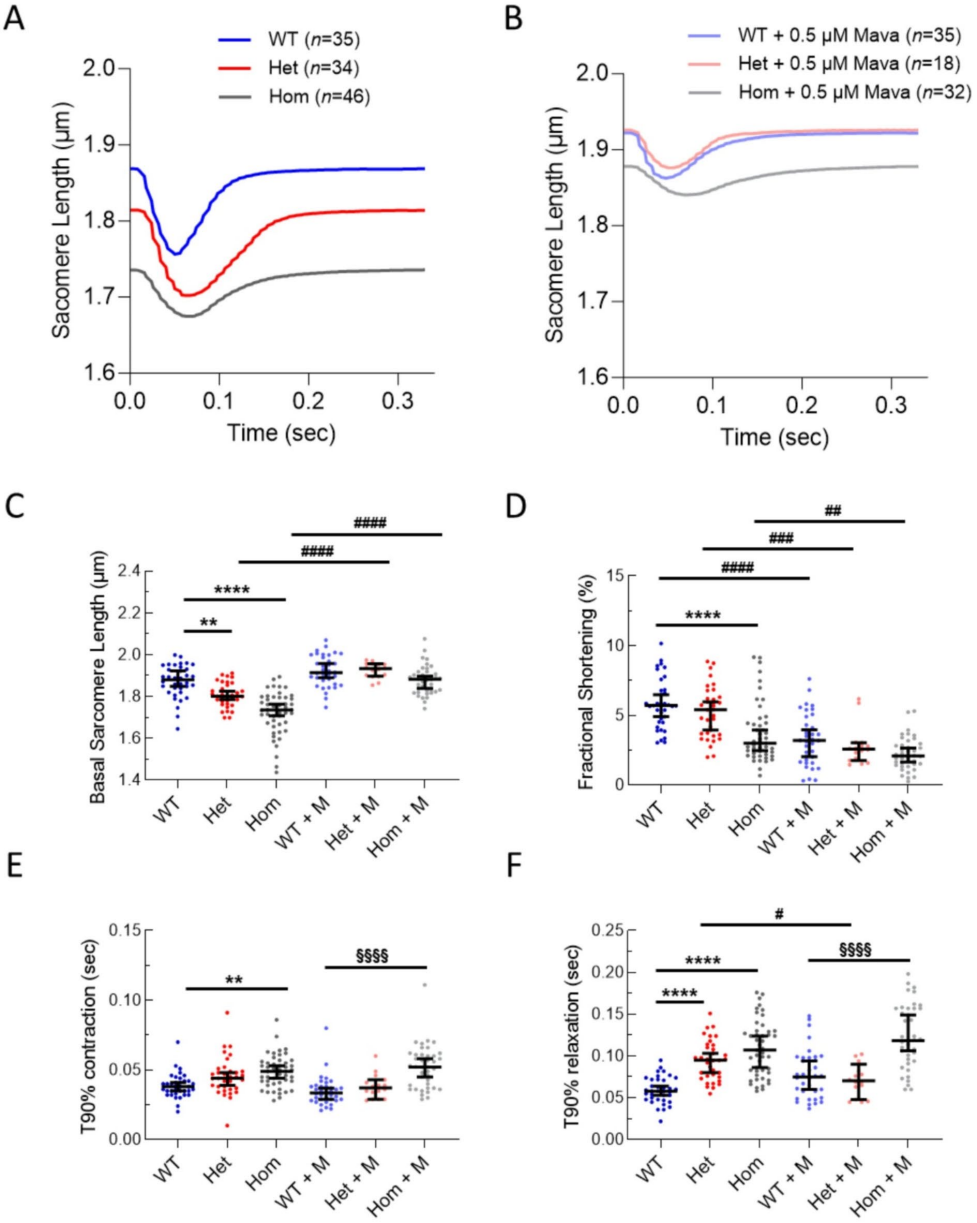
Isolated left ventricular cardiomyocytes from *Alpk3* K201X mice in the absence of the Ca^2+^ indicator fura2 displayed reduced diastolic sarcomere length, which is partially rescued by mavacamten treatment. (**A**) Average sarcomere length traces from heterozygous *Alpk3* K201X (Het, red) and homozygous *Alpk3* K201X cardiomyocytes (Hom, grey) decreased compared to wild type (WT) cardiomyocytes (blue). Contractile traces following treatment with 0.5 µM mavacamten (M) are shown in (**B**). Dot plots for selected extracted parameters: basal sarcomere length, fractional shortening, T90% contraction and T90% relaxation are given in (**C**–**F**) respectively. Data presented as median ± 95% confidence interval. Statistical differences comparing WT to Het and Hom are given by *, WT + M to Het + M and Hom + M are given by §, and control versus M for each genotype are given by #. They were calculated using a Kruskal-Wallis test with Dunns test for multiple comparisons., *p < 0.05, **p < 0.01, ***p < 0.001, ****p < 0.0001. Individual n numbers (cells per group of animals) are shown in (**A**) and (**B**) and all extracted parameters are tabulated in (Supplementary Table S6).

In isolated, unloaded cells, we observed a significant and dose-dependent decrease in basal sarcomere length in heterozygous and homozygous cells compared to WT cells (3.54 ± 0.52% and 8.06 ± 0.82%, respectively) and an increase in the time to 90% relaxation (T90_relaxation_) by (35.4 ± 4.1 ms and 49.4 ± 5.0 ms, respectively) for heterozygous and homozygous cells respectively compared to WT (Fig. 3A, C,F, S7, Table S6). Homozygous cells also showed a 10.3 ± 1.7 ms prolongation in contraction duration (Fig. 3A, E, S7, Table S6). These changes were also observed in the presence of calcium indicator fura2 (Figure S6, Table S6).

The reduction in fractional shortening for homozygous cells (Fig. 3D) was less robust and could not be replicated in cells loaded with fura2, where only heterozygous cells showed reduced fractional shortening (Fig. S6D). The discordance in the data may be attributable to contractile impairment caused by fura2^25^ and lower n numbers for dye-free measurements.

### Reduced protein kinase A dependent protein phosphorylation as a potential mechanism for contractile defects in *Alpk3* K201X cardiomyocytes

**Fig. 4:**
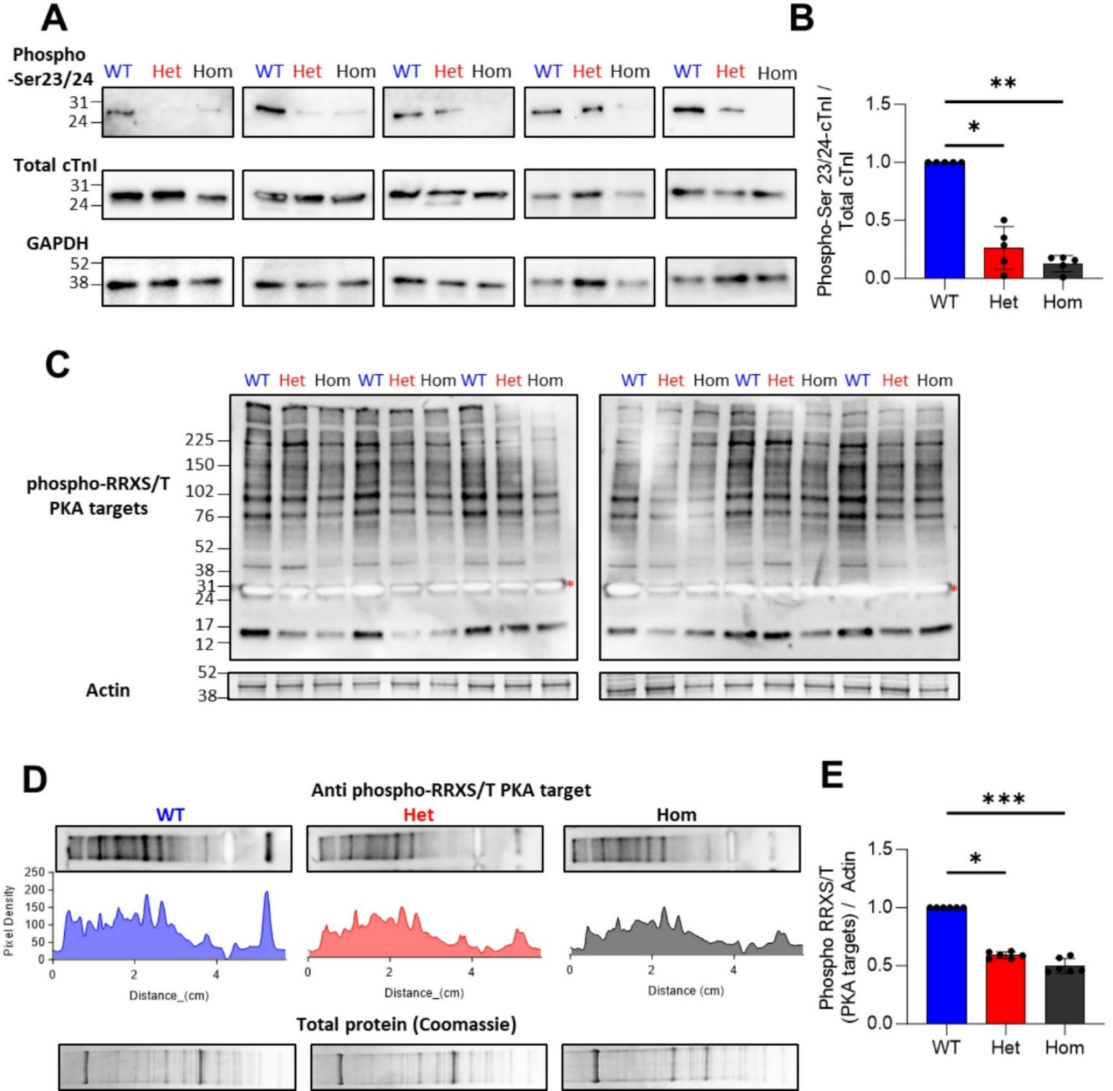
Depleted PKA dependent protein phosphorylation in *Alpk3* K201X mice Western blots of isolated cardiomyocyte preparations showed the phosphorylation of cTnI at Ser23/24 compared to total cTnI and GAPDH loading control (**A**), Densitometry analysis in (**B**) showed the degree of phosphorylation in wild type (WT), heterozygous (Het) and homozygous (Hom) mice (*n* = 5 mice per group). All PKA dependent phosphorylation targets were detected using an anti-phospho-RRXS/T antibody (**C** top), red stars denote an overexposed phospho-cTnI band, which was manually removed from the subsequent densitometric analysis. The loading control was cardiac actin, taken from the 42 kDa band on Coomassie stained control gels. (**D**) shows examples of lane densitometry measurements used to calculate cumulative band intensity across all detected proteins for WT, Het and Hom mice. The ratio of PKA target densitometry versus cardiac actin from Coomassie stained gels run in parallel is plotted in (**E**) (*n* = 6 mice per group). *p < 0.05, **p < 0.01, ***p < 0.001 using a Kruskal-Wallis test with Dunn’s test for multiple comparisons. Uncropped blots are shown in (Fig. S9).

In order to examine how a truncation in ALPK3, a protein with no known sarcomeric role, may be mediating an effect on contractility, we investigated the phosphorylation status of cardiac troponin I (cTnI). We found chronic reduction in serine23/24 cTnI modification (Fig. 4A, B), which is known to slow relaxation and sensitise the myofilament^26^. As this is a known site for phosphorylation by protein kinase A (PKA)^27^, we also assessed global PKA substrate status. We observed a dose-dependent reduction in all potential PKA target proteins (Fig. 4C). Cumulative densitometric analysis (Fig. 4D) and normalisation to a cardiac actin loading control showed that the level of PKA dependent phosphorylation is reduced by 39.3 ± 3.7% and 50.1 ± 2.6% in heterozygous and homozygous cells respectively (Fig. 4E).

### Abnormal calcium transients highlight complex changes to excitation-contraction coupling in cardiomyocytes from *Alpk3* K201X mice

**Fig. 5:**
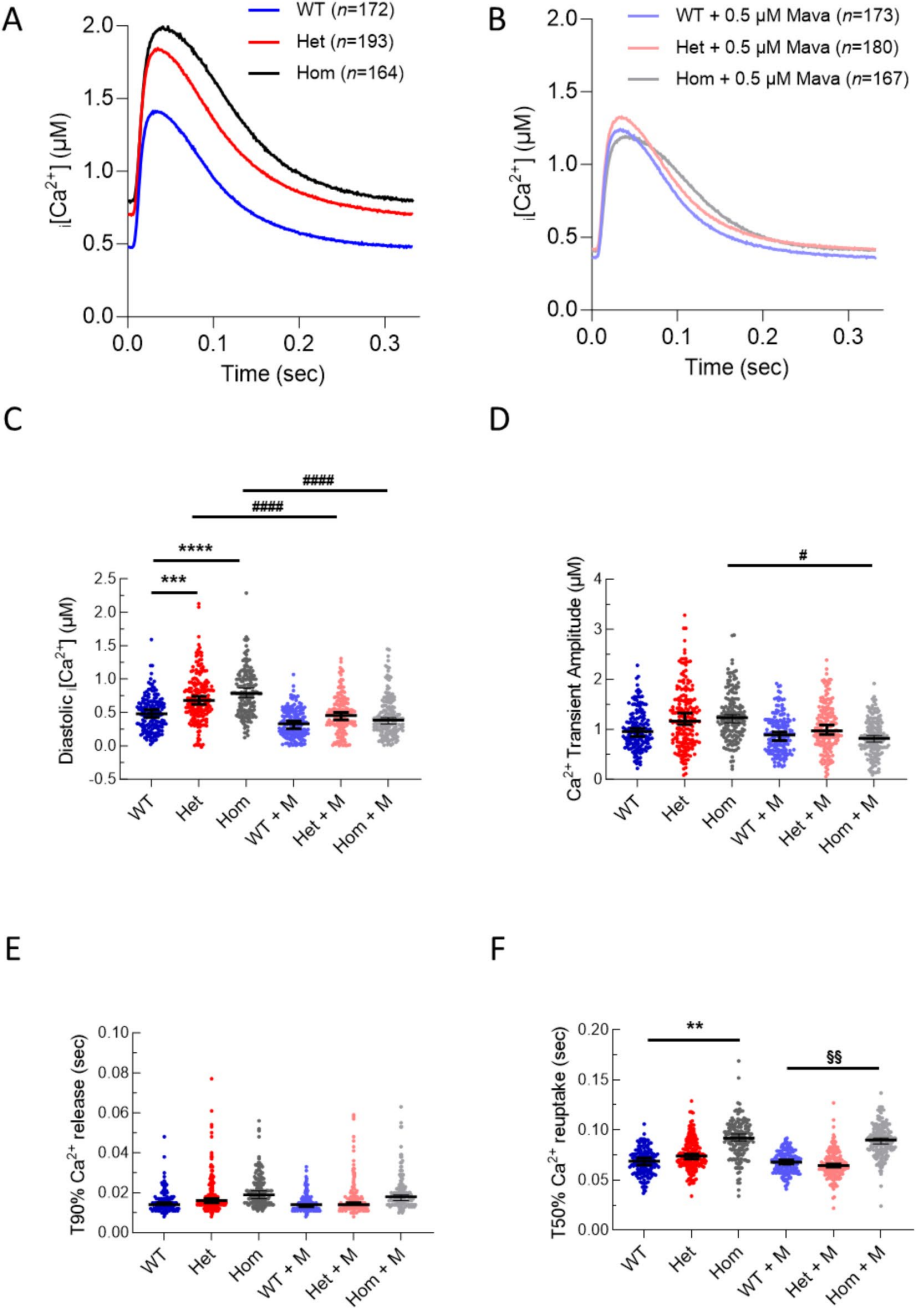
Isolated left ventricular cardiomyocytes from *Alpk3* K201X mice have elevated diastolic intracellular calcium levels and prolonged calcium reuptake, which is fully rescued by mavacamten treatment. (**A**) Average calibrated calcium transients from wild type (WT, blue), heterozygous *Alpk3* K201X (Het, red), homozygous *Alpk3* K201X cardiomyocytes (Hom, grey). (**B**) Calcium transients following treatment with 0.5 µM mavacamten (M) were similar between all genotypes. Dot plots for selected extracted parameters: Diastolic intracellular calcium levels [Ca^2+^]_i_ (**C**), calcium transient amplitude (**D**), time to 90% (T90%) calcium release (**E**) and time to 50% (T50%) calcium reuptake (**F**). Black lines represent median ± 95% confidence interval. Statistical differences comparing WT to Het and Hom are given by *, WT + M to Het + M and Hom + M are given by §, and control versus M for each genotype are given by #. They were calculated using a Kruskal-Wallis test with Dunns test for multiple comparisons, *p < 0.05, **p < 0.01, ***p < 0.001, ****p < 0.0001. Individual n numbers (cells per group of animals) are shown in (**A**) and (**B**) and all extracted parameters are tabulated in (Supplementary Table S6).

Calcium transients from fura2 loaded cardiomyocytes showed dose-dependent increases in diastolic intracellular calcium by ~ 50% for heterozygous and ~ 65% for homozygous mice compared to WT (Fig. 5A,C, S7, Table S6). These changes are concordant with reduced resting sarcomere length. Homozygous cells also showed an increase of 22.0 ± 1.5 ms in time to 50% reuptake (T50_reuptake_) (Fig. 5A,F, S7, Table S6). However, the calcium transient amplitude and Ca^2+^ release kinetics remained unchanged across all genotypes (Fig. 5A, D,E, S7, Table S6).

### Myosin conformation changes in *Alpk3* K201X mice are consistent with HCM

A reduction in the ratio of super-relaxed state (SRX) to disordered-relaxed state (DRX) of myosin heads is a molecular hallmark of sarcomeric HCM, observed in cardiac tissue from human patients and in animal models^28^. To probe whether mice with the *Alpk3* K201X variant have changes in the states of myosin heads, myosin ATP binding was investigated using cardiac tissue from WT, heterozygous and homozygous mice.

Heterozygous and homozygous mice showed a significantly reduced proportion of myosin heads in the SRX state, compared to WT littermates (Fig. S8). This suggests molecular changes occurring in the heterozygous *Alpk3* K201X mice are consistent with HCM.

### Mavacamten partially improves the cellular phenotype

Mavacamten is an allosteric myosin ATPase inhibitor with promising symptomatic improvements in HCM patients^29,30^. To test potential beneficial effects on our *Alpk3* K201X model, we treated isolated cardiomyocytes from our mouse model with 0.5 µM mavacamten and repeated contractility and calcium transient measurements to probe for a beneficial effect of mavacamten.

Treatment of WT cells showed reduced fractional shortening (Fig. 3A–D, Fig. S6A–D) with unchanged diastolic calcium levels and transient amplitude (Fig. 5A–D) as previously described^31,32^. When treating both heterozygous and homozygous *Alpk3* K201X cells, mavacamten rescued basal sarcomere length (Fig. 3C, S6C, S7, Table S6), concordant with reducing diastolic Ca^2+^ levels (Fig. 5C, S7, Table S6). However, it caused a reduction in fractional shortening also in these genotypes (Fig. 3D, S6D, S7, Table S6).

Mavacamten treatment did not improve contraction or relaxation time for homozygous cells (Fig. 3E, F, S6E, F, S7, Table S6) nor did it normalise Ca^2+^ reuptake time for homozygous cells (Fig. 5F, S7, Table S6).

A beneficial effect on heterozygous cells was found upon mavacamten treatment; relaxation time was normalised compared to WT (Fig. 3F, S6F, S7, Table S6), however this was not mirrored by changes in Ca^2+^ reuptake (Fig. 5F, S7, Table S6).

In summary, mavacamten can rescue elevated diastolic calcium levels in the mouse model and has beneficial effects on resting sarcomere length.

## Discussion

Using genetic screening, we confirmed that heterozygous *ALPK3tv* can cause autosomal dominant HCM. Case-control analysis revealed an excess of rare *ALPK3tv* in our HCM case cohort, compared to controls. The level of enrichment observed in our cohort (2.0%; OR 16.07, 95% CI 4.27–52.05) was comparable to that reported in recent studies (1.56%; OR 16.17, 95% CI 10.31–24.87)^9^. This indicates that while heterozygous *ALPK3tv* are causal for HCM, they have a lower odds ratio, reflecting lower penetrance, than seen with typical sarcomeric HCM genes. This is in keeping with the lack of overt HCM in the majority of the heterozygous carriers in the *ALPK3tv* families first described with recessive cardiomyopathy.

In this study we have generated a novel mouse model incorporating the *Alpk3* K201X variant based on a patient’s rare heterozygous *ALPK3tv* identified in our HCM cohort. This variant was chosen for its amino-terminal position, expected to produce (if at all) a truncated ALPK3 protein without the (pseudo-)kinase domain. Reduced transcript levels for *Alpk3* were observed in a dose-dependent manner, reaching significant reduction in homozygous mice (Fig. S1C). This argues for at least partial haplo-insufficiency through nonsense-mediated decay. In the absence of a working antibody for ALPK3, the consequences on Alpk3 protein levels and the potential presence of truncated protein remain unknown.

In the homozygous setting, the mouse model recapitulates observations of clinical reports of biallelic carriers of *ALPK3tv*, leading to severe, often lethal, paediatric onset cardiomyopathy^8,11,12^: The homozygous *Alpk3* K201X mice have impaired systolic function, dilatation and hypertrophy; we found their phenotype indistinguishable from *Alpk3* knockout mice. In our hands, both models survive well into adulthood. This is in contrast to a recent report on the same *Alpk3* knockout mice^2^, which – despite similar cardiac observations of systolic dysfunction and hypertrophy – did not survive beyond 14 weeks. We can only speculate whether genetic background (C57bl/6J versus C57bl/6 N) or environmental factors, e.g. diet, health status and microbiome^33^, could be responsible for the observed differences between laboratories.

Despite our aim to mirror the pathological human *ALPK3tv* found in our patient cohort, the heterozygous mice showed no overt cardiac phenotype at 3 or 6 months of age. This agrees with a recent report using an *Alpk3* null mouse model: mice with a heterozygous deletion of *Alpk3* developed hypertrophy only when aged beyond 1 year^2^. Future work will investigate whether our *Alpk3* K201X model will also develop late onset hypertrophy if aged for a similar timespan.

In addition, McNamara et al. reported normal cardiac dimensions and function in 3-week-old mice heterozygous for the c.5294G > A, p.W1765X *ALPK3* patient-specific variant, whereas mice homozygous for this variant presented with pronounced systolic and diastolic dysfunction as well as left ventricular hypertrophy^4^.

Moreover, due to differences in cardiac physiology and lack of stressors, the full disease spectrum of humans often cannot be fully reflected in animal models. This has also been reported for other heterozygous cardiomyopathy gene models, such as *Csrp3*,* Mybpc3*,* Myh7* and *Ttn* displaying phenotypes in homozygous settings only, whilst heterozygous animals showed no cardiac abnormalities^17,34–36^. Interestingly, individuals carrying heterozygous *ALPK3tv*, as family members of patients with recessive *ALPK3tv*, often have no clinical characteristics of HCM; only three out of 21 patients showed clinical signs of HCM^6,8,12^. Nevertheless, heterozygous *Alpk3* K201X mice showed an aggravated hypertrophic response to chronic adrenergic stimulation. This mirrors the observations of a *Mypbc3* HCM mouse model, where the same Iso/PE treatment helped to unmask disease features in the heterozygous mice^37^.

Despite the lack of baseline phenotype in vivo, isolated cardiomyocytes from both heterozygous and homozygous *Alpk3* K201X mice showed striking abnormalities: they displayed reduced diastolic sarcomere length, accompanied by an increase in diastolic calcium concentration. These are hallmarks of cardiomyocytes from sarcomeric HCM models^19,31^ and effects were dose-dependent, i.e. more pronounced in homozygous than in heterozygous *Alpk3* K201X cells.

In contrast to classical sarcomeric HCM models, the *Alpk3* K201X cells displayed some indications of reduced fractional shortening, usually observed in models of DCM^38^. While this fits with the systolic dysfunction observed in vivo for the homozygous mice, extrapolation from unloaded cardiomyocytes to whole organ contractility is uncertain, as factors such as disarray^30,39^ and myofilament density^40^ can influence whole organ contractile properties. Future work, using contractile measurements of skinned, loaded papillary muscle preparations exposed to different Ca^2+^ concentrations^41^, will help to address mechanistic questions of systolic dysfunction in the *Alpk3* K201X mouse model.

Moreover, we observed reduced PKA-mediated phosphorylation in *Alpk3* K201X cardiomyocytes, including markedly reduced phosphorylation of troponin I at serine23/24. The latter is predicted to result in increased troponin Ca^2+^ affinity and hence, at least in part, may be responsible for the observed slower relaxation and reduced sarcomere length^26^. Furthermore, reduced global PKA substrate phosphorylation may implicate changes to a range of other functional targets that may modulate contractility and Ca^2+^ handling^42^. Whether these observed functional changes are caused by ALPK3 directly regulating the activity and/or localisation of PKA and/or phosphodiesterases to alter phosphorylation levels, or are part of a chronic adaptation of the myofilaments remains unknown. In support of the latter, a phospho-proteome study using *ALPK3* mutant iPSC-derived cardiomyocytes cultured for a maximum of 30 days did not observe a reduction in phosphorylation of PKA-targets on the myofilament^4^.

Comparison of an acute (iPSC-cardiomyocyte) cellular model with the mouse model will also shed light on how complex remodelling of Ca^2+^ handling and excitation contraction coupling^43^ contribute to the unique molecular phenotypes of *ALPK3*-related cardiomyopathy. This may further help to address some apparent discrepancies in our current study, such as the potential indication of systolic dysfunction despite normal calcium transient amplitudes. The combination of experiments at different scales (employing myofilament, single cells, multi-cellular preparations) in acute and chronic settings will further allow dissection of a postulated causal role of altered PKA-signalling on Ca^2+^ handling, exciting contraction coupling and contractility in the presence of *ALPK3tv*.

Mavacamten is a myosin ATPase inhibitor which is now used to treat symptomatic obstructive HCM in patients^30^. Given the observed HCM-like dysfunction at cellular level, we tested whether mavacamten could restore calcium handling and contractility in the cardiomyocytes. Mavacamten rescued the reduced diastolic sarcomere length and increased diastolic calcium levels. This effect is most likely mediated through complex feedback loops between the myofilament cross bridge cycling and its Ca^2+^ sensitivity^19,43^. However, the drug had detrimental effects on fractional shortening in our cellular model, which parallels observations on HCM-causing variants in thin filament proteins, e.g. cardiac troponin T R92Q and cardiac troponin I R145G^31^.

Therefore, while we demonstrate here for the first time that mavacamten may have beneficial effects on patients with *ALPK3tv*, careful dose titration and tight surveillance might be required to prevent excessive reductions in systolic function in those patients. Future in vivo animal studies and clinical trials are needed to evaluate the benefits and risks of mavacamten treatment in this patient group.

In conclusion, our novel *Alpk3* K201X mouse model for HCM caused by autosomal dominant *ALPK3tv* demonstrates hallmarks of HCM at the level of isolated cardiomyocytes. To our knowledge, this is the first illustration of a ‘non-sarcomeric’ HCM disease gene manifesting such effects. Our findings further indicate that ALPK3 may be a modulator of PKA signalling, which may explain some of the complex molecular changes in the presence of *ALPK3tv*. We further demonstrate that mavacamten can restore some aspects of cellular dysfunction caused by *ALPK3tv* and may therefore be a therapeutic option for patients with *ALPK3tv*.

## Electronic supplementary material

Below is the link to the electronic supplementary material.

Supplementary Material 1

## Data Availability

Data underlying this article will be shared on reasonable request to the corresponding author.
