# Supplementary material for: An ALPK3 truncation variant causing autosomal dominant hypertrophic cardiomyopathy is partially rescued by mavacamten
Source: Sci Rep. 2025 Mar 24;15:10090. doi: 10.1038/s41598-025-94371-w (PMC11933305; doi:10.1038/s41598-025-94371-w)
Supplement: Supplementary file 1 — Supplementary Material 1 [file 41598_2025_94371_MOESM1_ESM.pdf]

An ALPK3 truncation variant causing autosomal dominant hypertrophic cardiomyopathy is partially rescued by mavacamten

Lisa Leinhos, Paul Robinson, Giulia Poloni, Sophie Broadway-Stringer, Julia Beglov, Adam B. Lokman, Gillian Douglas, Sajjad Nuthay, Oveena Fonseka, Manuel Schmid, Evie Singer, Charlotte Hooper, Kate Thomson, Richard D. Bagnall, Jodie Ingles, Christopher Semsarian, Elizabeth Ormondroyd, Christopher N Toepfer, Benjamin Davies, Charles Redwood, Hugh Watkins, Katja Gehmlich

Electronic Supporting Information

Supplemental material and methods

Alpk3 knock-out mice

Alpk3 knock-out (KO) mice ($Alpk3^{tm1b(EUCOMM)Hmgu}$) were obtained from EUCOMM, embryo-rederived and backcrossed onto C57BL/6JOlaHsd (Envigo RMS (UK) Ltd) for at least 6 generations until congenic before phenotyping.

Genotyping was performed using the REDExtract-N-Amp™ Tissue PCR Kit (Sigma, for primer pairs see below) and separate PCRs for the presence of the WT and the KO allele. PCR products were analysed by agarose electrophoresis (1.5% agarose in Tris-Borate-EDTA buffer, ThermoFisher Scientific).

WT allele (401 bp product)

Forward primer: 5’ – GGTCAAGACTCCATTCAGCCCT – 3’

Reverse primer: 5’ – CCTCCACGCCTATTCTAGCCTC – 3’

KO allele (358 bp product):

Forward primer: 5’ – GCGAGCTCAGACCATAACTT – 3’

Reverse primer: 5’ – CCCAAGTCACAAACTGTTCA – 3’

Quantitative Reverse Transcriptase Polymerase Chain Reaction (qPCR)

qPCRs were performed as described [1] using the Taqman probes (Applied Biosystems) listed in Table S7.

Cell measurements

Cardiomyocytes were isolated from the three genotypes and fixed (see Immunofluorescence). Individual cells were documented on bright field setting with an EVOS M5000 imaging system (ThermoFisher Scientific), using a 20x objective. In ImageJ 1.53e, length and width of individual rod-shaped cardiomyocytes were determined manually for each cell and recorded. For statistical analysis, ROUT outlier test was performed and outliers (1/118 in heterozygous and 1/163 in homozygous cells for length and 12/118 for Het and 1/163 for homozygous cells for width omitted). Data were analysed with nested 1-way ANOVA taking into consideration that cells were derived from 2 WT, 3 Het and 3 Hom mice.

Immunofluorescence

Snap frozen cardiac tissue was embedded in O.C.T compound mounting medium (VWR Chemicals) for cryotome processing (10 µm thick cryosections). Samples were rinsed in 1x PBS and permeabilized with 0.2% Triton X-100 in PBS for 5 min. After another rinse in 1x PBS, the cardiac samples were blocked in 10% normal goat serum in PBS for 1h at room temperature. Samples were incubated in primary antibody (diluted in 1% BSA Gold Buffer: 20 mM Tris-HCl pH 7.5; 155 mM NaCl; 2 mM EGTA; 2 mM $MgCl_2$) in a humidity chamber at 4°C overnight. Elisabeth Ehler (King's College London) kindly gifted the anti-MYOM1 (1:50) antibody (clone B4, raised in mouse) for localisation of myomesin. For α-actinin, anti-α-actinin2 antibody (1:50, EP2529Y, Abcam, rabbit) was used. Samples were rinsed in PBT (0.002% Triton X-100 in PBS) and washed three times for 5 min each in PBT, followed by secondary antibody incubation in a humidity chamber for 1h at room temperature. For secondary antibody conjugates, anti-mouse Alexa Fluor 488 (1:100, A11017, Life Tlife echnologies) and anti-rabbit Alexa Fluor 568 (1:100, A21144, Life Technologies) were used. After incubation, the samples were rinsed and washed three times in PBT for 5 min. Nuclei were counterstained using NucBlue (R37605, Invitrogen) according to the manufacturer's guidelines. Coverslips were mounted with mounting media. Samples were analysed with Leica DM 6000 CFS Fluorescent Microscope using a 63x HCX PL AP0 oil objective.

Isolated cardiomyocytes were spun onto poly-lysine coated slides, fixed with 4% paraformaldehyde in PBS for 10 min and processed on the slides, apart from incubation of both primary and secondary antibodies overnight. Images were recorded on a Zeiss LSM880 Confocal Microscope with X63 Plan-APOCHROMAT water immersion lens at room temperature, using Zen 2.3 software.

Images were processed and analysed with ImageJ software.

Histology

Paraffin-embedded hearts were cut into 7 µm thick sections. Samples were incubated in Histo-Clear and washed in 100% ethanol, followed by 95% ethanol for a few minutes. After rinsing in 1x PBS, sections were stained with hematoxylin for 30 s and rinsed under tap water before being counterstained with eosin for 30 s. After rinsing under tap water and in 1x PBS, samples were incubated in 95% ethanol and 100% ethanol. Before coverslips were applied, the samples were cleared of paraffin residues with Xylene. Images were taken using Axio Scan .Z1 slide scanner at 4x and 20x magnification in bright field mode using a 3CCD colour 2MP Hitachi 1200x1600 HV F202SCL camera.

Mant-ATP assay and analysis

The Mant-ATP assay was performed on murine myocardial samples using a protocol adapted from Toepfer et al. [2].

In brief, after defrosting in permeabilization buffer (100 mM NaCl 8 mM $MgCl_2$, 5 mM EGTA, 5 mM K_2HPO_4, 5 mM KH_2PO_4, 3 mM NaN_3, 5 mM ATP, 1 mM DTT, 20 mM 2,3-butanedione monoxime, BDM, 0.1% Triton-X 100 at pH 7.0), the permeabilization of samples took place for 6 hrs on a rocker with exchange of the solution every 2 hrs. Samples were dissected following storage at −20°C in glycerinating solution (120 mM K acetate, 5 mM Mg acetate, 2.5 mM K_2HPO_4, 2.5 mM KH_2PO_4, 50 mM MOPS, 5 mM ATP, 20 mM BDM, 2 mM DTT, 50% glycerol (v/v), pH 6.8.) overnight. The myocardial tissue was then processed into ~ 90 × 400 µm thin samples and placed in a chamber, followed by an additional permeabilization in permeabilization buffer for 10 min on ice and flushed using glycerinating buffer. The residual glycerol was replaced with rigor buffer (120 mM K acetate, 5 mM Mg acetate, 2.5 mM K_2HPO_4, 2.5 mM KH_2PO_4, 50 mM MOPS, 2 mM DTT at pH 6.8) for 10 min. Fluorescence acquisition to visualize fluorescent Mant-ATP wash-in was initiated by adding rigor buffer premixed with 250 µM Mant-ATP. Acquisition of the Mant-ATP chase was initiated after 10 min by the addition of ATP buffer (Rigor buffer + 4 mM ATP) to the chambers. A Nikon TE2000-E with a Nikon 20X/0.45 objective / Hamamatsu C9100 EM-CCD was used to visualize the fluorescence acquisition every 5 s / 20 ms over a time frame of 10 min in a DAPI filter setting. Mant-nucleotides were purchased from Invitrogen.

Fluorescence decay was measured in three independent areas per tissue sample. ImageJ ROI manager was used for the analysis. The subtraction of non-myosin bound Mant-ATP fluorescence signal was corrected by factor 52% as per [3], the y-intercept describes the final

fluorescence wash in data point. Data was normalized as initial fluorescent intensity and double exponential decay was calculated using the following equation:

$$\text{Normalized Fluorescence} = 1 - \text{A1}\ (1 - \exp^{(-\,t/T1)}) - \text{A2}\ (1 - \exp^{(-\,t/T2)})$$

The initial rapid decay is represented by A1, and after non-specific Mant-ATP correction approximates the proportion of the disordered-relaxed state (DRX) of myosin heads, where T1 is the time constant. A2 indicates the slower decay resembling the super-relaxed state (SRX) of myosin heads and T2 is the time constant respectively.

Supplemental tables and figures

Table S1: *ALPK3tv* detected in NIHR Bioresource Rare Disease HCM project (BRRD) cohort (HCM) and in GnomAD (controls).

Table S2: HCM case versus control analyses

Abbreviations echocardiography parameters

Table S3. Summary of echocardiography parameters of wildtype (WT), heterozygous (Het) and homozygous (Hom) *Alpk3 K201X* mice aged 3 months as well as WT and Het at 6 months.

Table S4. Summary of echocardiography parameters of homozygous global *Alpk3* knock out mice (KO) versus homozygous *Alpk3* K201X mice at 3 months.

Table S5. Summary of echocardiography parameters of wildtype (WT) and heterozygous (Het) *Alpk3 K201X* mice after chronic adrenergic challenge (Iso/PE) or sham saline treatment.

Table S6. Extracted parameters for unloaded sarcomere shortening and Ca^{2+} transient measurements from isolated left ventricular cardiomyocytes.

Table S7. Taqman Assays for qPCR

Figure S1. Position of *ALPK3tv* in our HCM cohort and generation of a novel mouse model *Alpk3* K201X

Figure S2. Morphological and functional comparison of the hearts

Figure S3. qPCR demonstrates induction of transcripts related to foetal gene programme and hypertrophic signalling in homozygous (Hom) *Alpk3* K201X Hom mice

Figure S4. Cellular hypertrophy, but no evidence of fibrosis in hearts of *Alpk3* K201X mice

Figure S5. *Alpk3* K201X mice show no evidence for dysregulated myomesin

Figure S6. Isolated left ventricular cardiomyocytes from *Alpk3* K201X mice in the presence of the Ca^{2+} indicator fura2 display reduced resting sarcomere length with prolonged relaxation

Figure S7. Cluster analysis illustration from all significant extracted parameters taken from unloaded sarcomere shortening and calcium transient measurements

Figure S8. Molecular changes in the heterozygous mice are consistent with HCM

Figure S9. Whole western blot images.

Table S1. *ALPK3tv* detected in NIHR Bioresource Rare Disease HCM project (BRRD) cohort (HCM) and in GnomAD (controls). Genomic co-ordinates according to genome buildGRCh38. Coding (c.) and protein (p.) nomenclature according to MANE transcripts (NM_929778.5; ENST0000025888.6). ACMG criteria and classification according to [4,5]. * Variants in last exon in the gene, may escape nonsense-mediated decay.

Genomic co-ordinates (chromosome 15)	c.	p.	Predicted effect	HCM/control	ACMG criteria	Classification
g.84817514del	c.62del	p.(Gly21Alafs*88)	Frameshift	HCM	PVS1	Likely pathogenic
g.84839880A>T	c.601A>T	p.(Lys201*)	Nonsense	HCM	PVS1; PM2	Likely pathogenic
g.84840483del	c.1204del	p.(Ala402Profs*14)	Frameshift	HCM	PVS1; PM2	Likely pathogenic
g.84856391G>A	c.1654-1G>A	p.?	Splice site	HCM	PVS1; PM2	Likely pathogenic
g.84862795C>G	c.4290C>G	p.(Tyr1430*)	Nonsense	HCM	PVS1; PM2	Likely pathogenic
g.84839076del	c.401del	p.(Arg134Profs*39)	Frameshift	Control	PVS1; PM2	Likely pathogenic
g.84840372C>T	c.1093C>T	p.(Gln365*)	Nonsense	2 x Controls	PVS1	Likely pathogenic
g.84856782G>T	c.2044G>T	p.(Glu682*)	Nonsense	Control	PVS1	Likely pathogenic
g.848857193C>T	c.2455C>T	p.(Arg819*)	Nonsense	Control	PVS1	Likely pathogenic
g.84862718C>T	c.4213C>T	p.(Arg1405*)	Nonsense	Control	PVS1	Likely pathogenic
g.84868446del	c.5108del	p.(Gly1703Alafs*53)	Frameshift**	Control	PVS1_moderate; PM2	VUS
g.84868453del	c.5115del	p.(*1706Serext*49)	Stop loss**	2 x Controls	PVS1_moderate	VUS

Table S2. HCM case versus control analyses using 230 HCM cases and 6,219 controls (see Material and Methods). FET=Fishers exact test. OR=Odds ratio. Lower CI=lower 95% confidence interval of OR. Upper CI=upper 95% Confidence interval of OR.

Variant type	HCM case frequency	Control frequency	FET p-value	OR	Lower_CI	Upper CI
**All variants**	0.0565	0.0263	0.0116	2.2147	1.1394	3.9596
**Missense**	0.0348	0.0243	0.2788	1.45	0.61	2.97
**Truncating**	0.0217	0.0014	**0.0001**	**16.07**	4.27	52.05
**Non-truncating**	0.0348	0.0250	0.3869	1.41	0.59	2.88

Abbreviations echocardiography parameters

**EF (%)**	Ejection fraction
**FS SAX (%)**	Fractional Shortening in short axis view
**LV Mass (mg)**	Left ventricle mass calculated by system
**LVAW:d (mm)**	Left ventricle anterior wall thickness in diastole
**LVAW:s (mm)**	Left ventricle anterior wall thickness in systole
**LVPW:d (mm)**	Left ventricular posterior wall thickness in diastole
**LVPW:s (mm)**	Left ventricular posterior wall thickness in systole
**LVID:d (mm)**	Left ventricular internal diameter in diastole
**LVID:s (mm)**	Left ventricular internal diameter in systole
**LV Vol:d (uL)**	Left ventricular volume in diastole
**LV Vol:s (uL)**	Left ventricular volume in systole
**PSV (mm/sec)**	Peak systolic velocity
**HR (/min)**	Heart rate
**HW/TL (mg/mm)**	Heart weight to tibia length

Table S3. Summary of echocardiography parameters of wildtype (WT), heterozygous (Het) and homozygous (Hom) *Alpk3 K201X* mice aged 3 months as well as WT and Het at 6 months.

Data shown as mean ± SEM. Where not all animals of a genotype could be analysed, the number of animals analysed for this parameter stated in brackets. Statistical significance indicated as for Hom versus WT at 3 months; ** p < 0.01, *** < p<0.001, **** p < 0.001; Kruskal-Wallis test. No significant changes were observed for Het versus WT at either age (Student's t-test at 6 months).

**Age**	**3 months**				**6 months**	
	**WT**	**Het**	**Hom**		**WT**	**Het**
**N**	23	21	8		10	9
**EF (%)**	66±2	61±2	28±3 ****		67±3	66±4
**FS SAX (%)**	36.5±1.7	37±3	12.9±1.5 ****		37± 2	37±3
**LV Mass (mg)**	117±7	124±6	157±8 **		118±5	125±9
**LVAW:d (mm)**	0.91±0.05	0.95±0.04	1.01±0.07		0.99±0.05	1.07±0.09
**LVAW:s (mm)**	1.38±0.07	1.34±0.07	1.18±0.09		1.24±0.06	1.40±0.08
**LVPW:d (mm)**	0.99±0.06	0.91±0.05	1.06±0.05		0.90±0.05	0.88±0.03
**LVPW:s (mm)**	1.45±0.05 (20)	1.29±0.07 (18)	1.25±0.04		1.4±0.06	1.36±0.11
**LVID:d (mm)**	3.91±0.07	4.03±0.09	4.42±0.09 **		3.970±0.07	4.00±0.10
**LVID:s (mm)**	2.50±0.10	2.73±0.11	3.86±0.11****		2.50± 0.12	2.55±0.17
**LV Vol:d (uL)**	67±3	72±4	89±4 **		69±3	71±4
**LV Vol:s (uL)**	24±2	29±3	65±4 ****		23 ±3	25±3
**PSV (mm/sec)**	19.8±0.9 (22)	19.6±0.9	12.2±1.1 ***		21.4±0.9	20.7±0.5
**HR (/min)**	478±6 (20)	469±5	488±4		466±13	486±12
**HW/TL (mg/mm)**	7.4±0.3 (16)	8.5±0.3 (9)	13.1±0.2 **** (16)		8.2±0.2	8.9±0.3

Table S4. Summary of echocardiography parameters of homozygous global *Alpk3* knock out mice (KO) versus homozygous *Alpk3* K201X mice at 3 months.

Data shown as mean ± SEM. Number of animals per genotype and parameter stated in brackets. No statistical differences in the phenotypes of the two models were observed, apart from minor difference in heart rate (* $p < 0.05$; Student's t-test used).

Please note the *Alpk3* K201X (Hom) cohort is a different cohort to the one shown in Fig. 1 and Table S3.

	***Alpk3* KO**	***Alpk3* K201X (Hom)**
**N**	13	13
**EF (%)**	30.6±1.8	36±4
**FS SAX (%)**	14.4±0.9	15.6±1.4 (12)
**LV Mass (mg)**	166±7	177±9
**LVAW:d (mm)**	1.03±0.04	1.12±0.05
**LVAW:s (mm)**	1.14±0.03	1.16±0.05
**LVPW:d (mm)**	1.03±0.04	1.05±0.05
**LVPW:s (mm)**	1.17±0.04	1.33±0.09
**LVID:d (mm)**	4.57±0.09	4.55±0.08
**LVID:s (mm)**	3.92±0.10	3.76±0.13
**LV Vol:d (uL)**	97±5	95±4
**LV Vol:s (uL)**	67±4	62±4
**HR (/min)**	471±3	480±3 *
**HW/TL (mg/mm)**	12.6±0.4	13.3±0.3

Table S5. Summary of echocardiography parameters of wildtype (WT) and heterozygous (Het) *Alpk3 K201X* mice after chronic adrenergic challenge (Iso/PE) or sham saline treatment.

Data shown as mean ± SEM. Statistical significance indicated as * p < 0.05, 2-way-ANOVA test with Tukey's post-hoc test for multiple comparisons used. Sample size (N) in table refers to animals per group, for HW/TL n numbers are stated in brackets if different.

$^{\$}$ p < 0.05 versus WT Iso/PE,

* p < 0.05, ** p < 0.01, *** p < 0.001, **** p < 0.0001 versus Het sham,

$^{\#}$ p < 0.05, $^{\#\#\#}$ p < 0.001 versus WT sham

	sham		Iso/PE	
	**WT**	**Het**	**WT**	**Het**
**N**	12	8	10	12
**EF (%)**	67±5	65±5	72±4	68±3
**FS SAX (%)**	38±3	36±3	41±3	38±2
**LV Mass (mg)**	104±6	108±8	125±6	157±8***,$^{\$}$
**LVAW:d (mm)**	0.96±0.05	0.86±0.05	1.02±0.05	1.17±0.05***
**LVAW:s (mm)**	1.24±0.06	1.24±0.07	1.53±0.08$^{\#}$	1.60±0.07**
**LVPW:d (mm)**	0.95±0.04	0.99±0.08	1.03±0.04	1.06±0.04
**LVPW:s (mm)**	1.41±0.10	1.37±0.07	1.58±0.07	1.53±0.06
**LVID:d (mm)**	3.65±0.09	3.84±0.11	3.84±0.14	4.10±0.12
**LVID:s (mm)**	2.28±0.15	2.48±0.17	2.28±0.19	2.55±0.12
**LV Vol:d (uL)**	57± 3	64±4	65±5	75±5
**LV Vol:s (uL)**	19±3	23±4	20±4	24±3
**PSV (mm/sec)**	20.2±0.7	21.1±1.9	21.8±1.5	22.7±1.2
**HR(/min)**	482±4	472±6	539±26	549±24*
**HW/TL (mg/mm)**	8.4±0.3	8.7±0.3 (10)	9.97±0.16$^{\#\#\#}$ (11)	11.1±0.3****,$^{\$}$ (10)

Table S6. Extracted parameters for (**A**) unloaded, dye-free sarcomere shortening, (**B**) Ca^{2+} transient, and (**C**) unloaded fura2-loaded sarcomere shortening measurements from left ventricular cardiomyocytes isolated from WT, heterozygous (Het) and homozygous (Hom) *Alpk3* K201X cardiomyocytes, with and without treatment with 0.5 µM mavacamten.

Values are given as mean ± SEM. Exact sample size (n) per condition (cells per group of mice) is given in the table.

A

	(*n*) mice	(*n*) cells	Basal Sarcomere Length (µm)	Peak Sarcomere Length (µm)	Peak Height (µm)	Fractional Shortening (%)	T Peak Contraction	T10% Contraction	T50% Contraction	T90% contraction	T10% Relaxation (sec)	T50% Relaxation (sec)	T90% Relaxation (sec)
**WT**	3	35	1.872±0.014	1.758±0.015	0.114±0.006	6.779±0.312	0.051±0.002	0.012±0.001	0.024±0.001	0.039±0.001	0.011±0.001	0.026±0.001	0.059±0.003
**WT + 0.5 µM mava**	3	35	1.922±0.013	1.858±0.013	0.066±0.008	3.283±0.319	0.050±0.003	0.014±0.001	0.022±0.002	0.035±0.002	0.013±0.001	0.037±0.002	0.068±0.005
**Het**	2	34	1.806±0.010	1.716±0.008	0.091±0.003	6.910±0.321	0.064±0.004	0.016±0.001	0.031±0.001	0.044±0.001	0.025±0.001	0.057±0.004	0.095±0.004
**Het + 0.5 µM mava**	2	15	1.926±0.010	1.872±0.015	0.054±0.007	2.790±0.368	0.052±0.003	0.013±0.001	0.025±0.002	0.038±0.002	0.012±0.002	0.030±0.003	0.071±0.005
**Hom**	4	46	1.721±0.015	1.656±0.014	0.065±0.006	3.765±0.321	0.066±0.002	0.014±0.001	0.029±0.001	0.049±0.002	0.018±0.001	0.047±0.003	0.109±0.005
**Hom + 0.5 µM mava**	4	39	1.878±0.011	1.836±0.012	0.042±0.004	2.253±0.191	0.071±0.003	0.016±0.001	0.032±0.002	0.052±0.003	0.021±0.001	0.054±0.003	0.124±0.006

B

	(*n*) mice	(*n*) cells	Diastolic $_i[Ca^{2+}]$ (µM)	Systolic $_i[Ca^{2+}]$ (µM)	Ca^{2+} Transient Amplitude (µM)	T Peak Release (sec)	T10% Ca_{2+} Release (sec)	T50% Ca_{2+} Release (sec)	T90% Ca_{2+} Release (sec)	T10% Ca_{2+} Reuptake (sec)	T50% Ca_{2+} Reuptake (sec)	T90% Ca_{2+} Reuptake (sec)
**WT**	6	157	0.483±0.020	1.485±0.041	1.002±0.032	0.025±0.001	0.005±0.001	0.009±0.001	0.015±0.001	0.021±0.001	0.069±0.001	0.170±0.003
**WT + 0.5 µM mava**	6	164	0.332±0.018	1.237±0.035	0.905±0.030	0.025±0.001	0.006±0.001	0.008±0.001	0.015±0.002	0.024±0.001	0.068±0.001	0.156±0.003
**Het**	8	192	0.719±0.026	1.963±0.064	1.244±0.047	0.030±0.001	0.009±0.001	0.011±0.001	0.019±0.001	0.023±0.001	0.076±0.001	0.186±0.003
**Het + 0.5 µM mava**	8	182	0.464±0.021	1.506±0.045	1.042±0.035	0.028±0.001	0.007±0.001	0.010±0.001	0.017±0.001	0.022±0.001	0.066±0.001	0.161±0.003
**Hom**	5	164	0.798±0.027	2.067±0.051	1.269±0.038	0.034±0.001	0.008±0.001	0.011±0.001	0.021±0.001	0.032±0.001	0.091±0.001	0.192±0.003
**Hom + 0.5 µM mava**	5	167	0.442±0.024	1.261±0.036	0.819±0.032	0.033±0.001	0.008±0.001	0.013±0.001	0.022±0.001	0.033±0.001	0.089±0.001	0.181±0.003

C

	(*n*) mice	(*n*) cells	Basal Sarcomere Length (µm)	Peak Sarcomere Length (µm)	Peak Height (µm)	Fractional Shortening (%)	T Peak Contraction	T10% Contraction	T50% Contraction	T90% contraction	T10% Relaxation (sec)	T50% Relaxation (sec)	T90% Relaxation (sec)
**WT**	6	157	1.877±0.006	1.787±0.007	0.090±0.004	4.779±0.195	0.046±0.001	0.010±0.001	0.021±0.001	0.034±0.001	0.011±0.001	0.028±0.001	0.072±0.003
**WT + 0.5 µM mava**	6	164	1.955±0.004	1.920±0.005	0.035±0.002	1.802±0.109	0.042±0.001	0.011±0.001	0.021±0.002	0.033±0.001	0.011±0.001	0.029±0.001	0.071±0.004
**Het**	8	192	1.795±0.005	1.727±0.008	0.068±0.003	3.788±0.166	0.055±0.003	0.014±0.001	0.025±0.001	0.041±0.001	0.014±0.001	0.035±0.001	0.096±0.003
**Het + 0.5 µM mava**	8	182	1.901±0.004	1.872±0.005	0.029±0.002	1.540±0.094	0.050±0.002	0.013±0.001	0.024±0.001	0.038±0.002	0.012±0.001	0.031±0.001	0.085±0.003
**Hom**	5	164	1.736±0.005	1.663±0.005	0.074±0.003	4.065±0.158	0.084±0.002	0.017±0.001	0.033±0.001	0.059±0.001	0.027±0.001	0.065±0.002	0.134±0.003
**Hom + 0.5 µM mava**	5	167	1.850±0.005	1.829±0.005	0.022±0.001	1.165±0.074	0.069±0.003	0.018±0.001	0.033±0.002	0.053±0.003	0.019±0.001	0.055±0.002	0.126±0.004

Table S7. Taqman Assays for qPCR (all Applied Biosystems, FAM-MGB unless stated otherwise)

Transcript	*Species*	*Assay ID*	*Comments*
Acta1	Mouse	Mm00808218_g1	
Alpk3	Mouse	Mm00475369_m1	
Ankrd1	Mouse	Mm00496512_m1	
Ankrd2	Mouse	Mm00508030_m1	
Col1a1	Mouse	Mm00801666_g1	
Fhl1	Mouse	Mm04204611_g1	
Gapdh	Mouse	4352339E	VIC-MGB labelled
Myh7	Mouse	Mm00600555_m1	
Nppa	Mouse	Mm01255748_g1	
Nppb	Mouse	Mm01255770_g1	
Rcan1.4	Mouse	Mm00627762_m1	

Figure S1. Position of *ALPK3tv* in our HCM cohort and generation of a novel mouse model *Alpk3* K201X.

(**A**) **Positions of the heterozygous *ALPK3tv***, identified in adult onset HCM patients. Coding exons are numbered and positions of domains are indicated by colour. *ALPK3* K201X is printed in bold.

(**B**) **Sanger sequencing traces of the mouse model.** Top trace from a wildtype (WT) mouse and bottom trace from heterozygous *Alpk3* K201X mouse (Het) are shown. The codon 201 is indicated in blue (AAG coding for lysine, Lys, in WT and TAG coding for a Stop codon in Het). Two silent modifications are also indicated: one creates a *HindIII* restriction site in the Het, the other disrupts the PAM sequence.

(**C**) **Transcript levels of *Alpk3* in the novel mouse model *Alpk3* K201X.** *Alpk3* transcript was measured in WT (blue), *Alpk3* K201X Het (red) and *Alpk3* K201X Hom (grey) and Alpk3 knockout (KO, white) hearts relative to *Gapdh* by qPCR. Data was not normally distributed (Shapiro-Wilk test), hence Kruskal-Wallis test with Dunn's post hoc test was used. *Alpk3* was found to be reduced in Hom and KO mice with * $p < 0.05$ and *** $p < 0.001$, respectively. N numbers: WT 6, Het 6, Hom 5, KO 6.

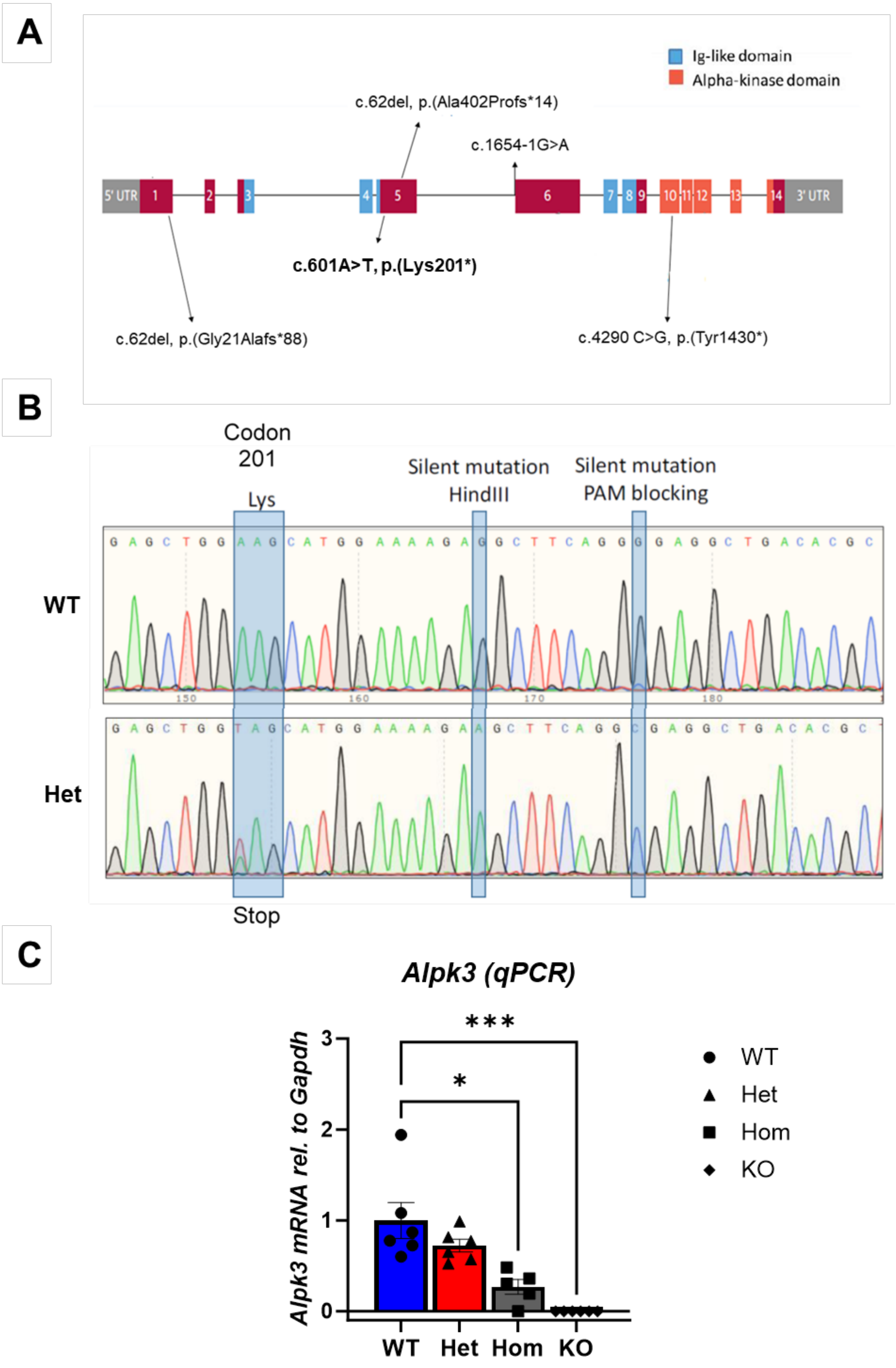
A
Ig-like domain
Alpha-kinase domain
c.62del, p.(Ala402Profs*14)
c.1654-1G>A
5' UTR
1
2
3
4
5
6
7
8
9
10
11
12
13
14
3' UTR
c.601A>T, p.(Lys201*)
c.62del, p.(Gly21Alafs*88)
c.4290 C>G, p.(Tyr1430*)
B
Codon 201
Lys
Silent mutation HindIII
Silent mutation PAM blocking
WT
Het
Stop
C
Alpk3 (qPCR)
Alpk3 mRNA rel. to Gapdh
0
1
2
3
***
*
WT
Het
Hom
KO

Figure S2. Morphological and functional comparison of the hearts

(**A**) Representative images of haematoxylin-eosin stained, long-axis heart sections of WT, *Alpk3* K201X Het and *Alpk3* K201X Hom mice to demonstrate the cardiac morphology.

(**B**) Representative examples of echocardiography (M-Mode) of WT, *Alpk3* K201X Het and *Alpk3* K201X Hom.

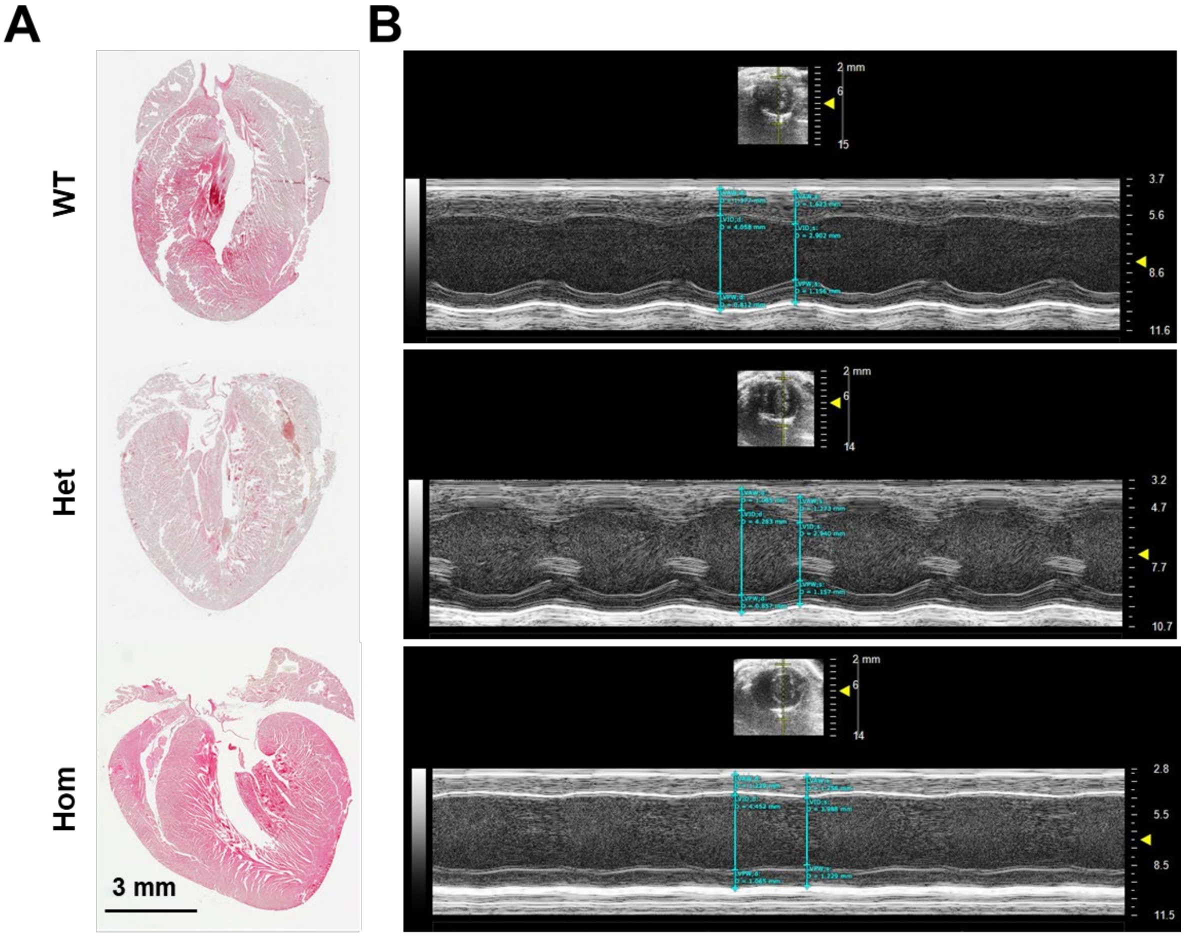

Figure S3. qPCR demonstrates induction of transcripts related to foetal gene programme and hypertrophic signalling in homozygous (Hom) *Alpk3* K201X mice.

Transcripts related to the foetal gene programme (**A**) and hypertrophic signalling (**B**) were measured in WT (blue), *Alpk3* K201X Het (red) and *Alpk3* K201X Hom (grey) and Alpk3 knockout (KO, white) hearts relative to Gapdh by qPCR. No induction as observed for Het mice, while induction of foetal gene programme and hypertrophic signalling was observed for Hom and KO mice. Data were normally distributed (Shapiro-Wilk test) for all but *Ankrd1* and *Fhl1*. For normally distributed data, 1-way-ANOVA with Dunnett's post hoc test was applied; for Ankrd1 and Fhl1 Kruskal Wallis test with Dunn's post hoc test was used. * $p < 0.05$, ** $p < 0.01$, *** $p < 0.001$, **** $p < 0.0001$ versus WT. N numbers: (A) WT 5, Het 6, Hom 4, KO 6; (B) WT 6, Het 6, Hom 5, KO 6.

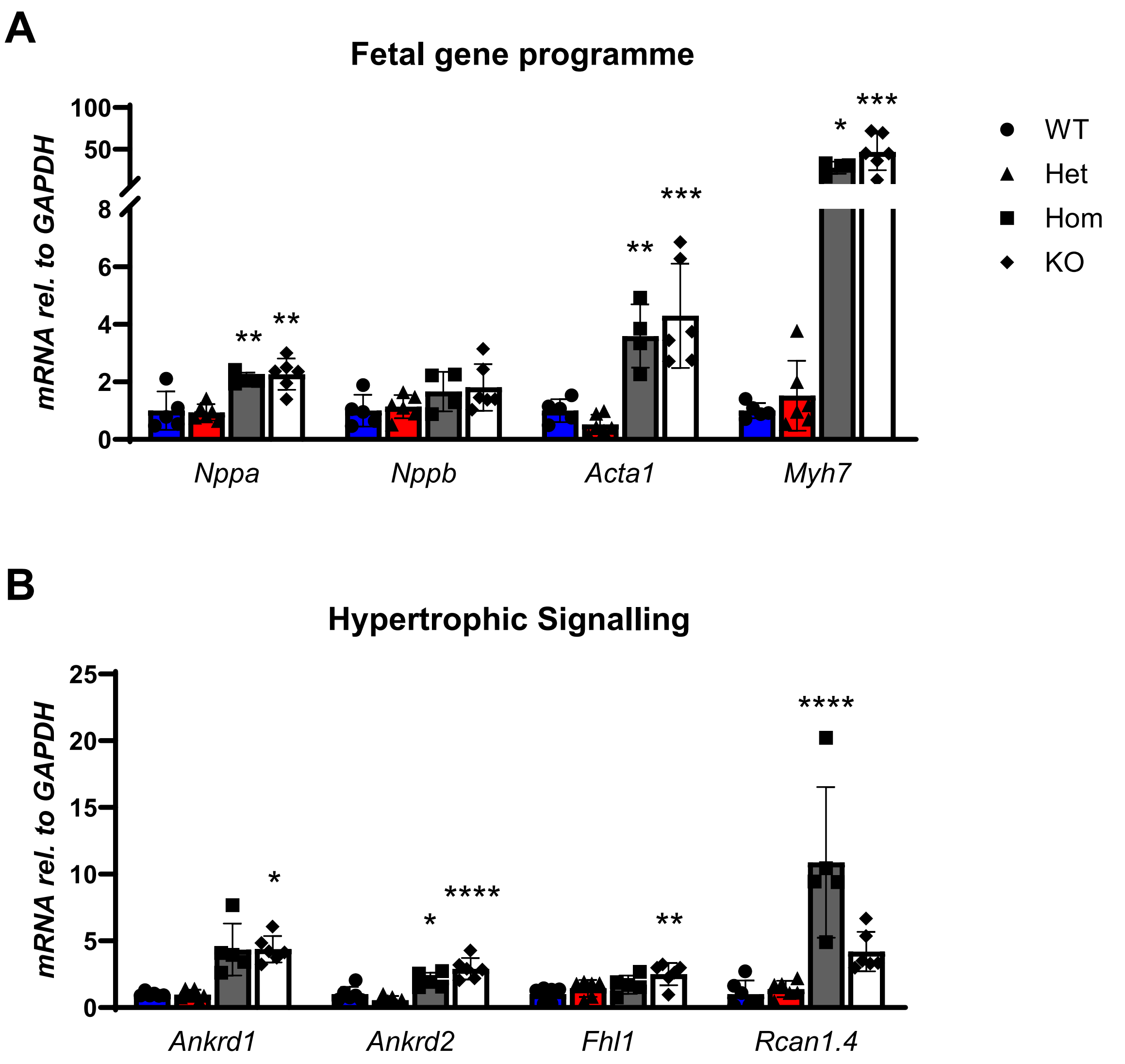

Figure S4. Cellular hypertrophy, but no evidence of fibrosis in hearts of *Alpk3* K201X mice

(**A**) **Histology of *Alpk3* K201X is unremarkable.** Representative images of haematoxylin-eosin stained, cardiac sections of wild type (WT), heterozygous (Het) *Alpk3* K201X and homozygous (Hom) *Alpk3* K201X mice shows no striking abnormalities. The same is true for homozygous *Alpk3* knockout mice (KO). Scale bare represents 50 microns.

(**B**) **No molecular evidence of fibrosis in heart *Alpk3* K201X mice.** Collagen *Col1a1* transcript was measured in WT (blue), *Alpk3* K201X Het (red) and *Alpk3* K201X Hom (grey) and Alpk3 knockout (KO, white) hearts relative to *Gapdh* by qPCR. Data was not normally distributed (Shapiro-Wilk test), hence Kruskal-Wallis test with Dunn's post hoc test was used. No evidence of induction of *Col1a1* was found in any of the genotypes, suggesting no induction of fibrosis. N numbers: WT 6, Het 6, Hom 5, KO 6.

(**C**) **Increased length, but not width in cardiomyocytes isolated from *Alpk3* K201X mice.** Isolated fixed cardiomyocytes from WT (blue), heterozygous (Het, red) and homozygous (Hom, grey) *Alpk3* K201 mice were measured for length and width. Length was increased in Het and Hom cells, * $p < 0.05$, *** $p < 0.001$, nested 1-way ANOVA. 86 WT cells from 2 mice, 117 Het cells from 3 mice and 162 cells from 3 mice were measured for length; 86 WT cells from 2 mice, 106 Het cells from 3 mice and 162 cells from 3 mice were measured for width.

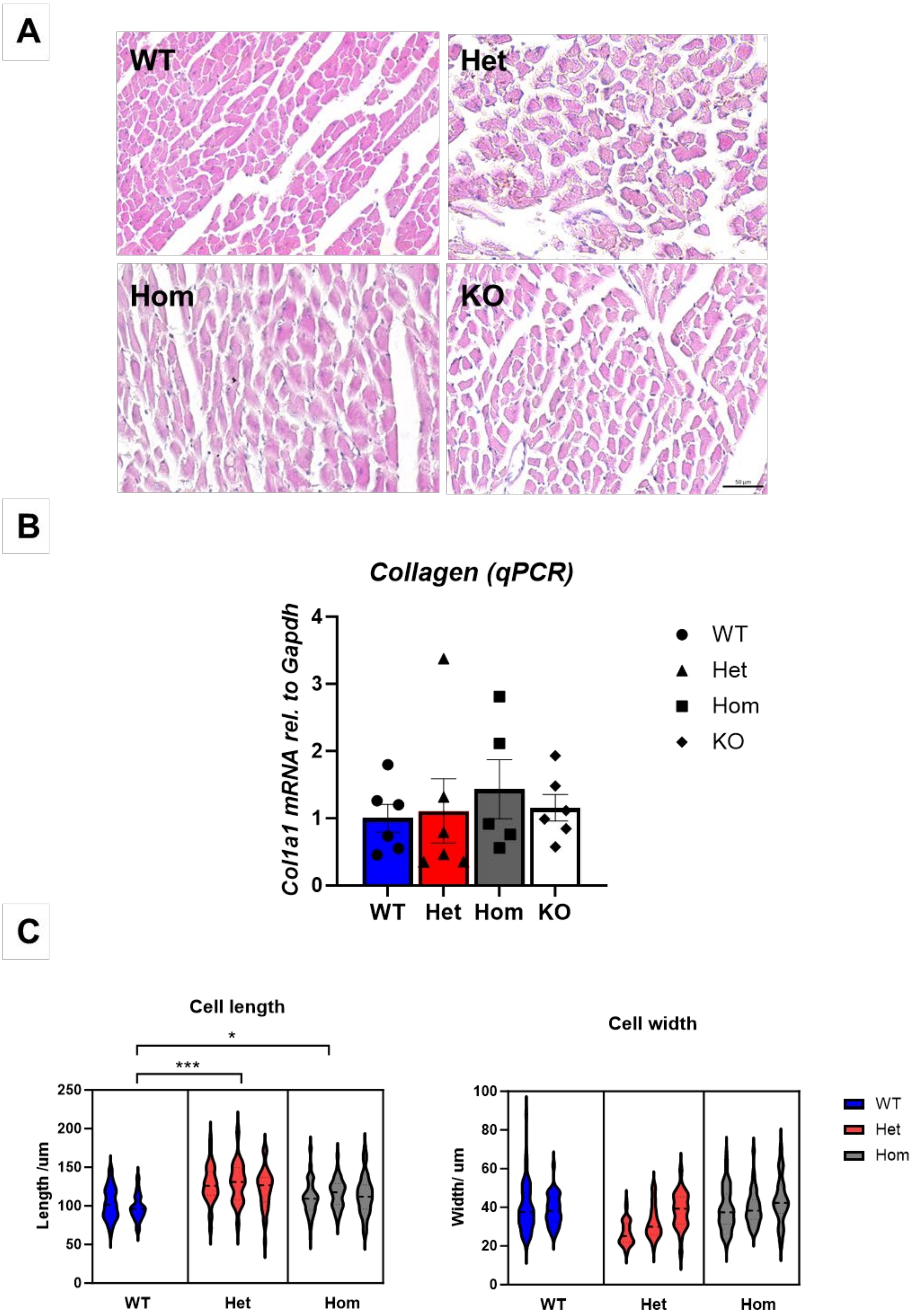

A
WT
Het
Hom
KO
50 µm
B
Collagen (qPCR)
Col1a1 mRNA rel. to Gapdh
0
1
2
3
4
WT
Het
Hom
KO
C
Cell length
*
***
Length /um
0
50
100
150
200
250
WT
Het
Hom
Cell width
Width/ um
0
20
40
60
80
100
WT
Het
Hom

Figure S5. ***Alpk3*** **K201X mice show no evidence for dysregulated myomesin**

(**A**) Cardiac tissue of heterozygous (Het) and homozygous (Hom) for *Alpk3 K201X* as well as a wildtype (WT) control was stained for M-band protein myomesin (green), alpha-actinin 2 (red) and nuclei (NucBlue, blue). Representative immunofluorescence images of each genotype are shown. Scale bar equals 25 µm and 10 µm for inset. Sections from at least three animals per genotype (n = 3) were processed.

(**B**) Isolated cardiomyocytes of Het and Hom *Alpk3 K201X* mice well as a WT cells were stained for myomesin and alpha-actinin as above (but without visualisation of nuclei). Scale bar equals 10 µm. Slides with isolated cells from two animals per genotype (n=2) were processed.

No changes of myomesin such as accumulation or abundance were detectable.

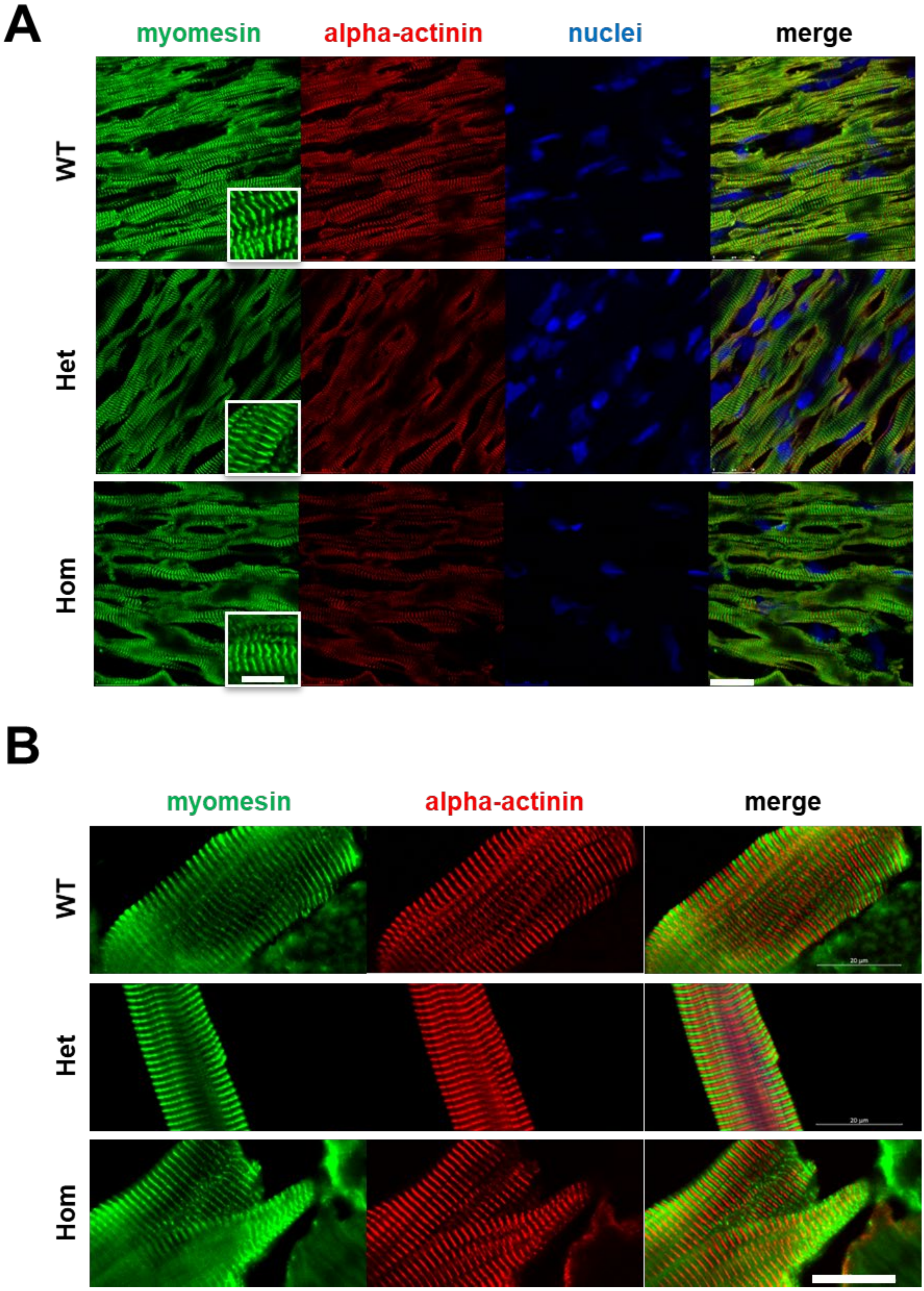
A
myomesin
alpha-actinin
nuclei
merge
WT
Het
Hom
B
myomesin
alpha-actinin
merge
WT
Het
Hom

Figure S6. Isolated, unloaded left ventricular cardiomyocytes from *Alpk3* K201X mice in the presence of the Ca^{2+} indicator fura2 display reduced sarcomere length with prolonged relaxation. (**A**) Average sarcomere length traces from WT (blue), heterozygous (Het, red) and homozygous *Alpk3* K201X (Hom, grey) cardiomyocytes without fura2 loading. (**B**) Contractile traces following treatment with 0.5 µM mavacamten are shown in B. Dot plots for selected extracted parameters: basal sarcomere length (**C**), fractional shortening (**D**), T90% contraction (**E**) and T90% relaxation (**F**), respectively. Black lines represent median ± 95% confidence interval. Sample size (n) is representing cells per condition and is indicated. Statistical differences comparing WT to Het and Hom are given by *, WT+M to Het+M and Hom+M are given by §, and control versus M for each genotype are given by #. They were calculated using a Kruskal-Wallis test with Dunns test for multiple comparisons. * $p < 0.05$, ** $p < 0.01$, *** $p < 0.001$, **** $p < 0.0001$. Individual n numbers (cells per group of animals) are shown in (**A**) and (**B**) and all extracted parameters are tabulated in supplementary Table S6.

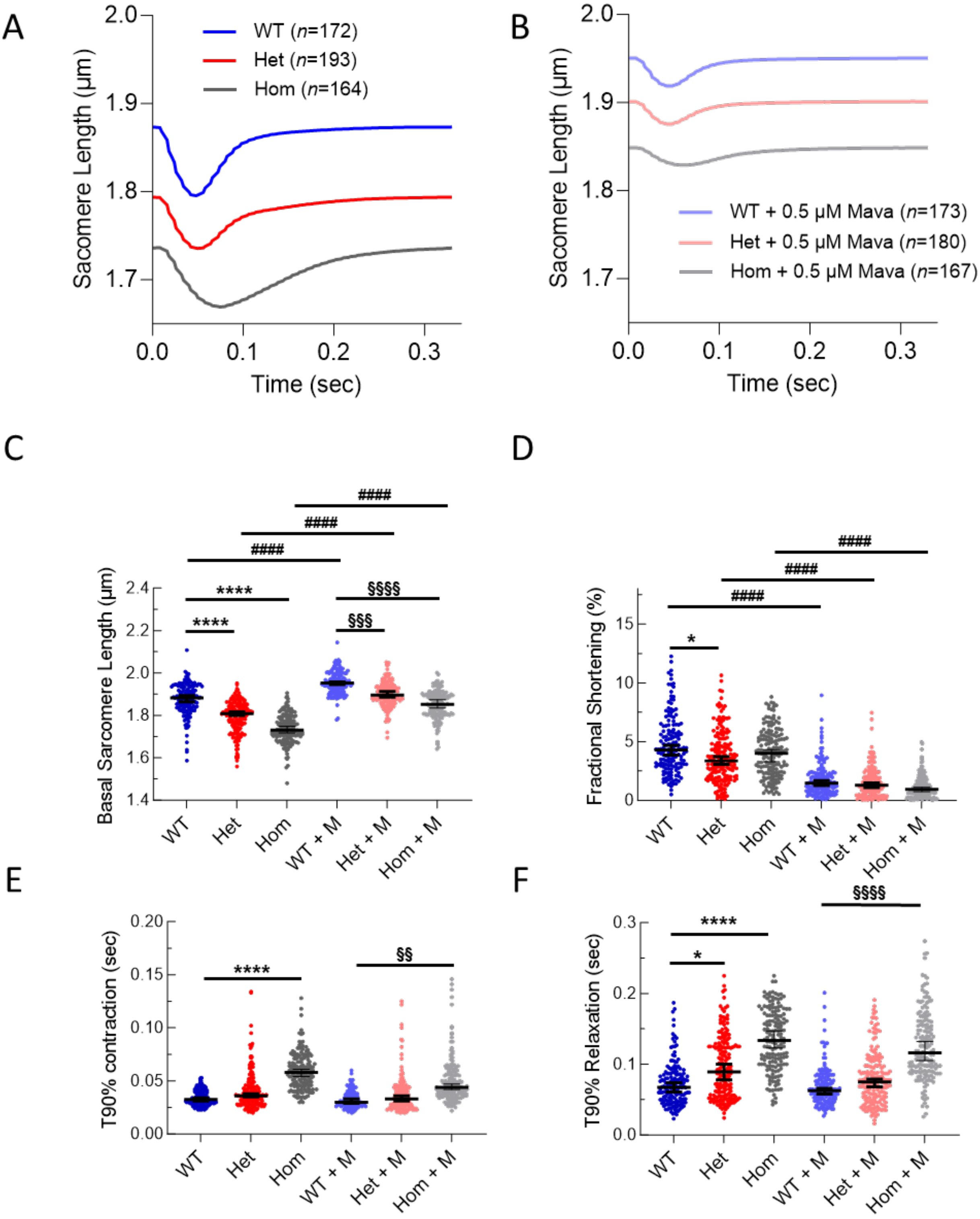
A
B
C
D
E
F
WT (n=172)
Het (n=193)
Hom (n=164)
WT + 0.5 µM Mava (n=173)
Het + 0.5 µM Mava (n=180)
Hom + 0.5 µM Mava (n=167)
Sacomere Length (µm)
Time (sec)
Basal Sarcomere Length (µm)
Fractional Shortening (%)
T90% contraction (sec)
T90% Relaxation (sec)
WT
Het
Hom
WT + M
Het + M
Hom + M

Figure S7. Cluster analysis illustration from key extracted parameters taken from unloaded sarcomere shortening and calcium transient measurements. Dots plots show parameters extracted from each cardiomyocyte split by individual mice for WT (blue), heterozygous (Het, red) and homozygous *Alpk3* K201X (Hom, grey) cardiomyocytes with and without treatment with 0.5 μM mavacamten. Parameters extracted include, basal sarcomere length (**A**), peak sarcomere length (**B**), fractional shortening (**C**), time to peak contraction (**D**), time to 50% contraction (**E**), time to 90% contraction (**F**), time to 50% relaxation (**G**), time to 90% relaxation (**H**), diastolic $[Ca^{2+}]_i$ (**I**), systolic $[Ca^{2+}]_i$ (**J**), Ca^{2+} transient amplitude (**K**), time to peak Ca^{2+} release (**L**), time to 90% Ca^{2+} release (**M**), time to 10% Ca^{2+} re-uptake (**N**), time to 50% Ca^{2+} re-uptake (**O**) and time to 90% Ca^{2+} re-uptake (**P**). Black lines are the median error bars are 95% confidence interval. Individual n numbers and all extracted parameters are tabulated in supplementary Table S6.

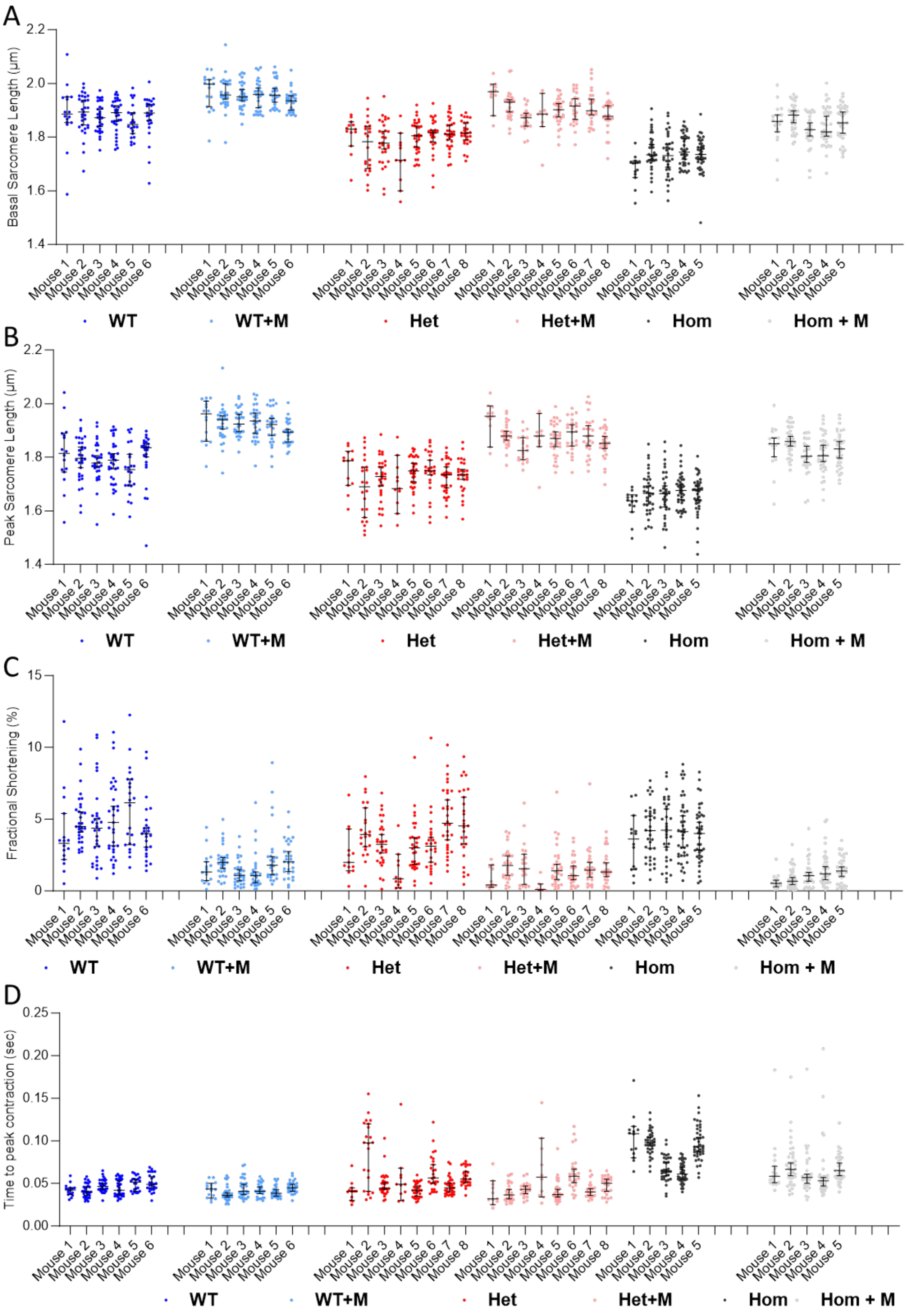
A
Basal Sarcomere Length (μm)
2.2
2.0
1.8
1.6
1.4
B
Peak Sarcomere Length (μm)
C
Fractional Shortening (%)
15
10
5
0
D
Time to peak contraction (sec)
0.25
0.20
0.15
0.10
0.05
0.00
Mouse 1
Mouse 2
Mouse 3
Mouse 4
Mouse 5
Mouse 6
Mouse 7
Mouse 8
WT
WT+M
Het
Het+M
Hom
Hom + M

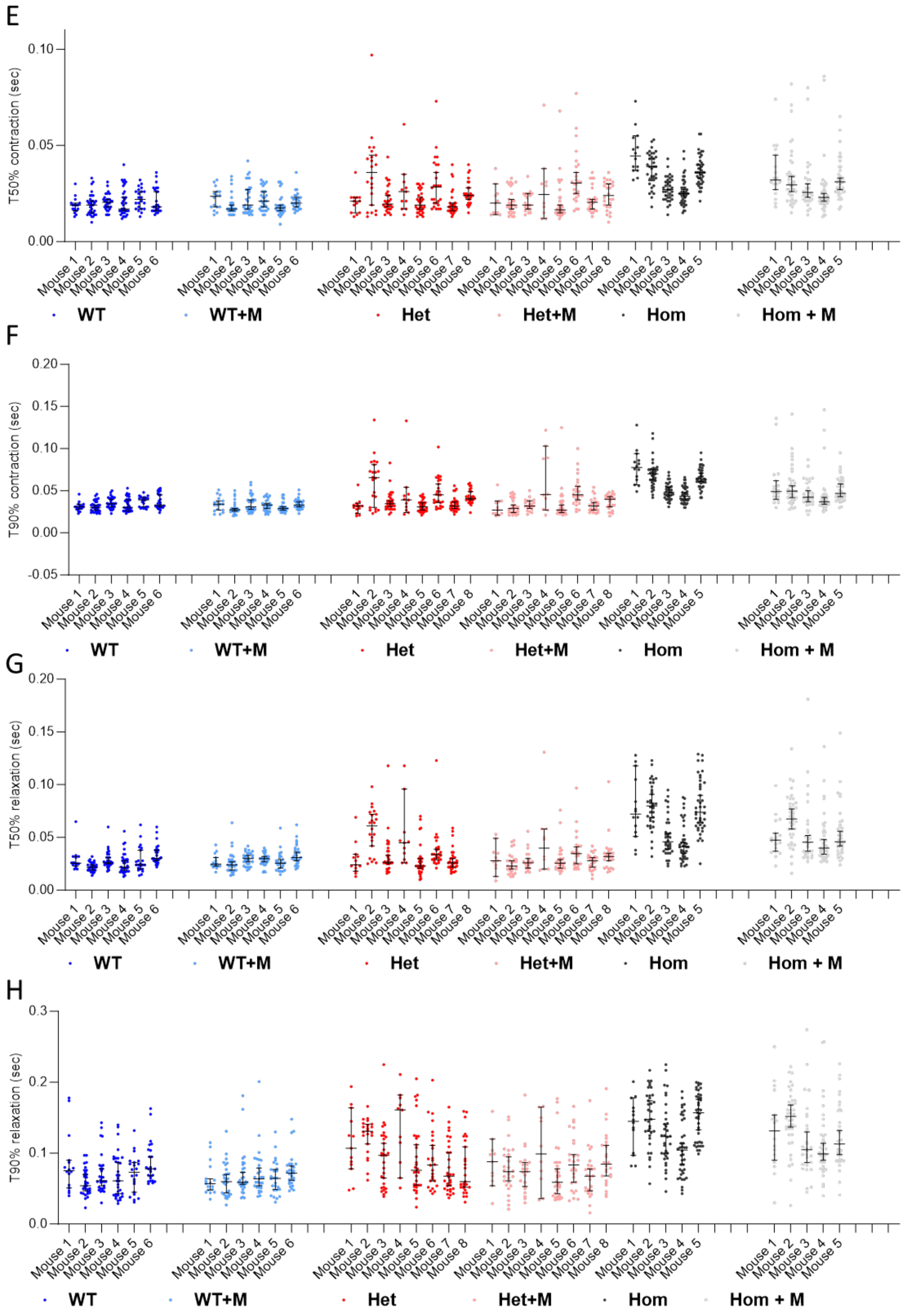
E
T50% contraction (sec)
0.10
0.05
0.00
Mouse 1
Mouse 2
Mouse 3
Mouse 4
Mouse 5
Mouse 6
Mouse 7
Mouse 8
WT
WT+M
Het
Het+M
Hom
Hom + M
F
T90% contraction (sec)
0.20
0.15
0.10
0.05
0.00
-0.05
G
T50% relaxation (sec)
H
T90% relaxation (sec)
0.3
0.2
0.1
0.0

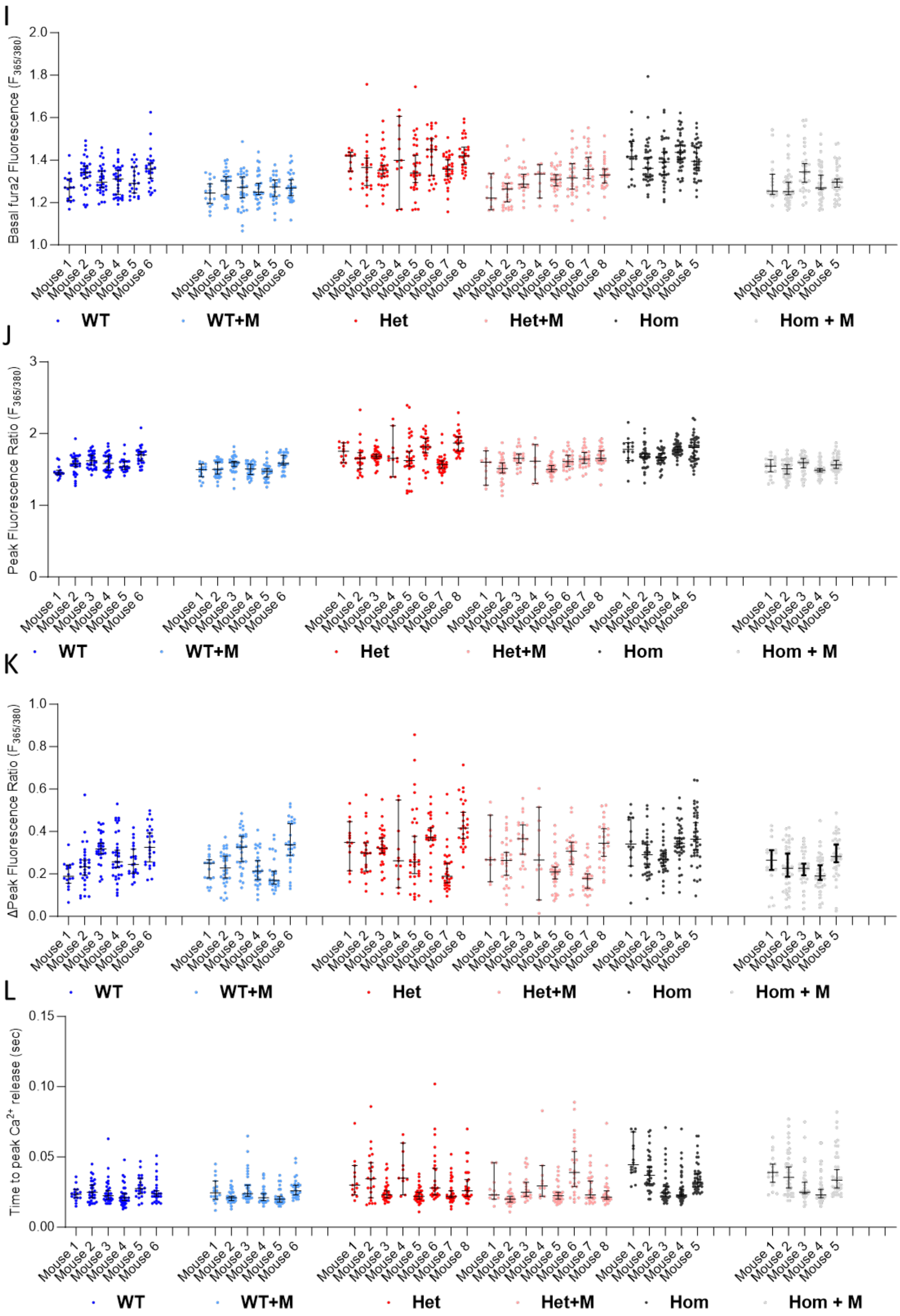
I
Basal fura2 Fluorescence (F365/380)
2.0
1.8
1.6
1.4
1.2
1.0
Mouse 1
Mouse 2
Mouse 3
Mouse 4
Mouse 5
Mouse 6
Mouse 7
Mouse 8
WT
WT+M
Het
Het+M
Hom
Hom + M
J
Peak Fluorescence Ratio (F365/380)
3
2
1
0
K
ΔPeak Fluorescence Ratio (F365/380)
1.0
0.8
0.6
0.4
0.2
0.0
L
Time to peak Ca2+ release (sec)
0.15
0.10
0.05
0.00

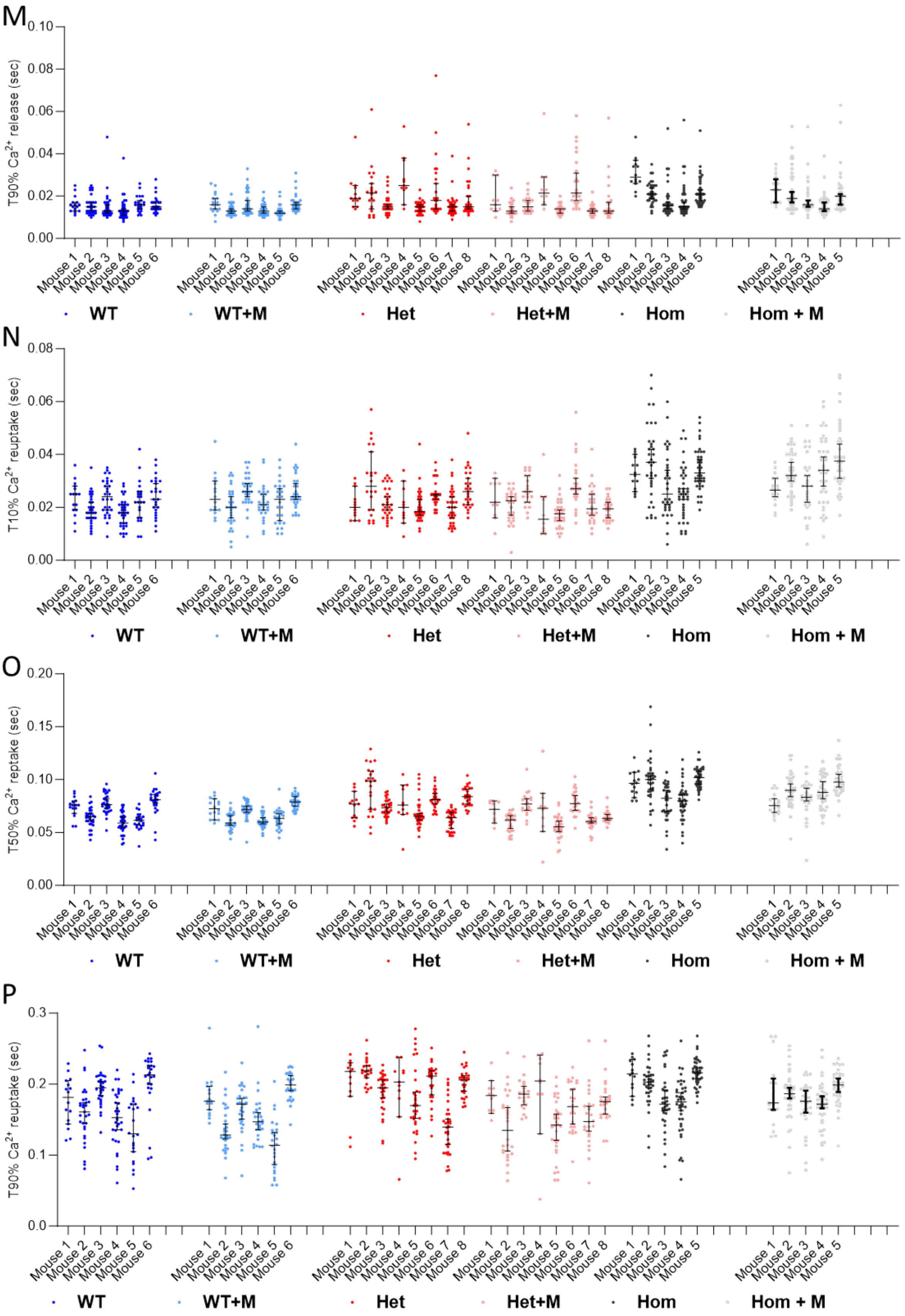
M
T90% Ca2+ release (sec)
0.00
0.02
0.04
0.06
0.08
0.10
Mouse 1
Mouse 2
Mouse 3
Mouse 4
Mouse 5
Mouse 6
Mouse 7
Mouse 8
WT
WT+M
Het
Het+M
Hom
Hom + M
N
T10% Ca2+ reuptake (sec)
O
T50% Ca2+ reptake (sec)
0.05
0.10
0.15
0.20
P
T90% Ca2+ reuptake (sec)
0.0
0.1
0.2
0.3

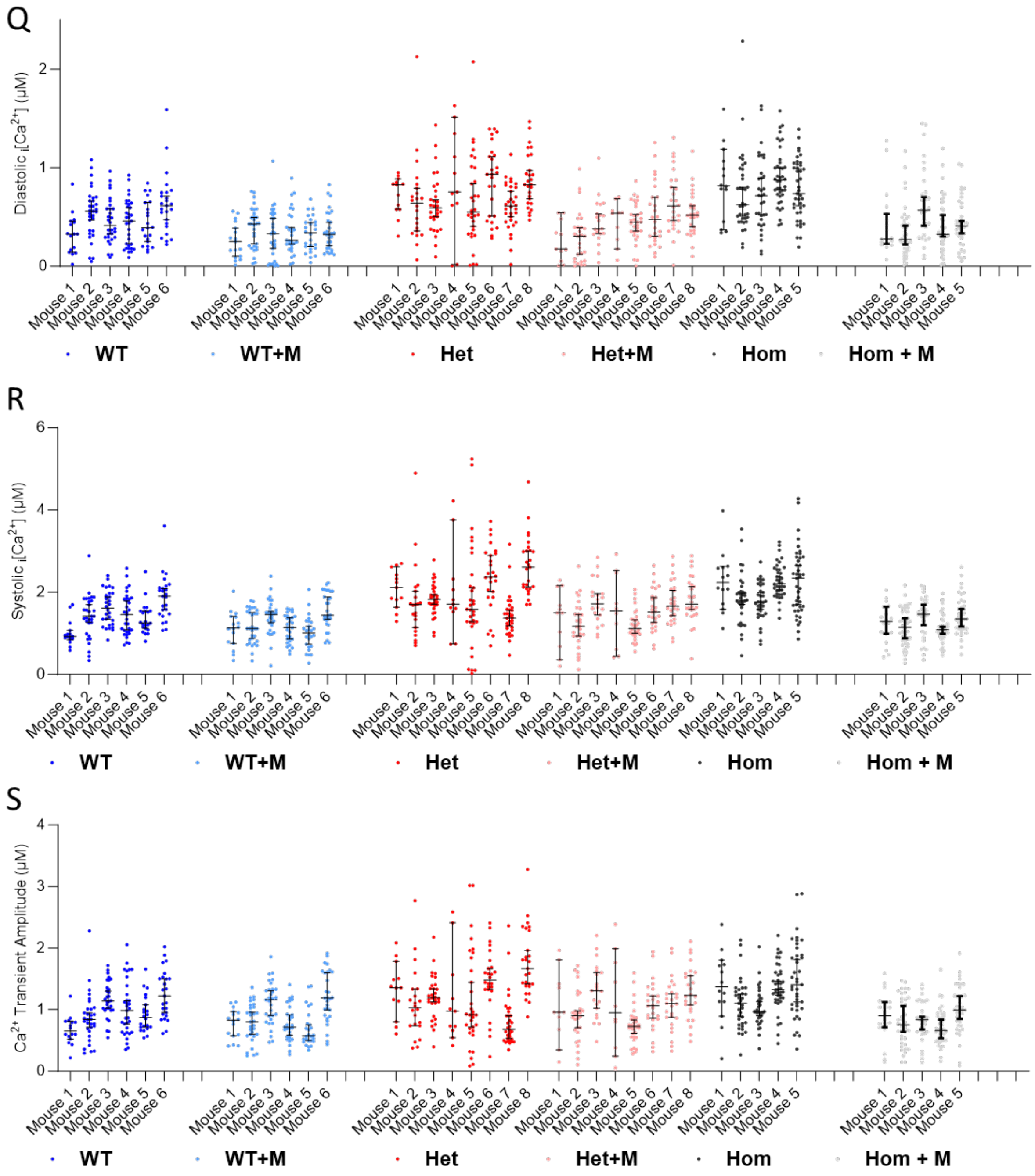
Q
Diastolic $_i$[Ca^{2+}] (µM)
Mouse 1
Mouse 2
Mouse 3
Mouse 4
Mouse 5
Mouse 6
Mouse 7
Mouse 8
WT
WT+M
Het
Het+M
Hom
Hom + M
R
Systolic $_i$[Ca^{2+}] (µM)
S
Ca^{2+} Transient Amplitude (µM)

Figure S8. Molecular changes in the heterozygous mice are consistent with HCM. Myosin ATP binding was analysed via a Mant-ATP assay on cardiac tissue of 3-month-old *Alpk3 K201X* mice. (**A**) Averaged curves of Mant-ATP hydrolysis for cardiac tissue from wildtype (WT), heterozygous (Het) and homozygous (Hom) *Alpk3 K201X* mice. (**B**) Despite the absence of cardiac functional or structural changes on echocardiography Het *Alpk3 K201X* mice showed a significantly reduced proportion of myosin heads in the super-relaxed state (SRX), compared to WT littermates. This was also observed in Hom animals. Values are presented as mean ± SEM. ** $p < 0.01$, *** $p < 0.001$ versus WT (Kruskal-Wallis test, n=4-6 technical replicates, n=3 biological replicates per genotype).

A

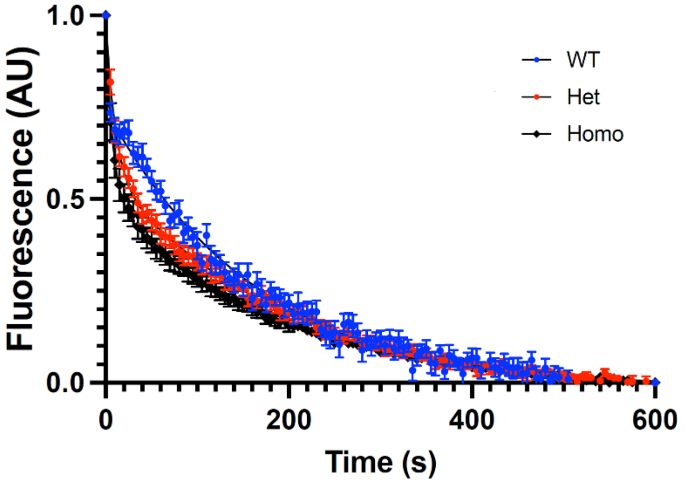

B

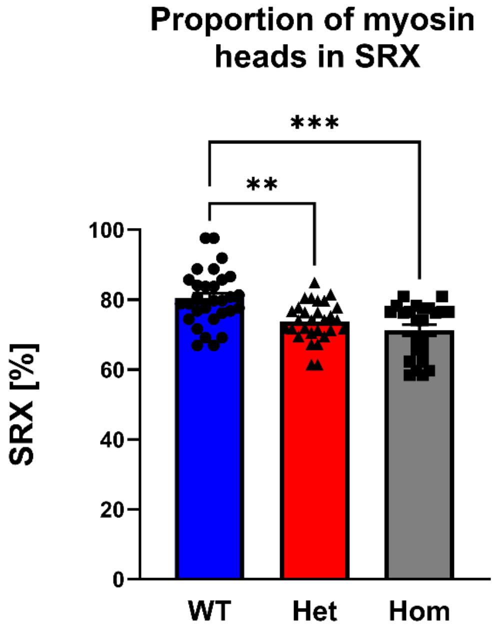

Figure S9. Whole western blot images. Images show whole western blots that were zoomed (red boxes) to create **Figure 4A and C (blots in A and B respectively).** Red star in **B (top)** highlights overexposed / photobleached phospho-cTnI, red star in **B (bottom)** denotes BSA contaminant from cellular isolation. Both bands were manually removed from lane scan densitometry calculations.

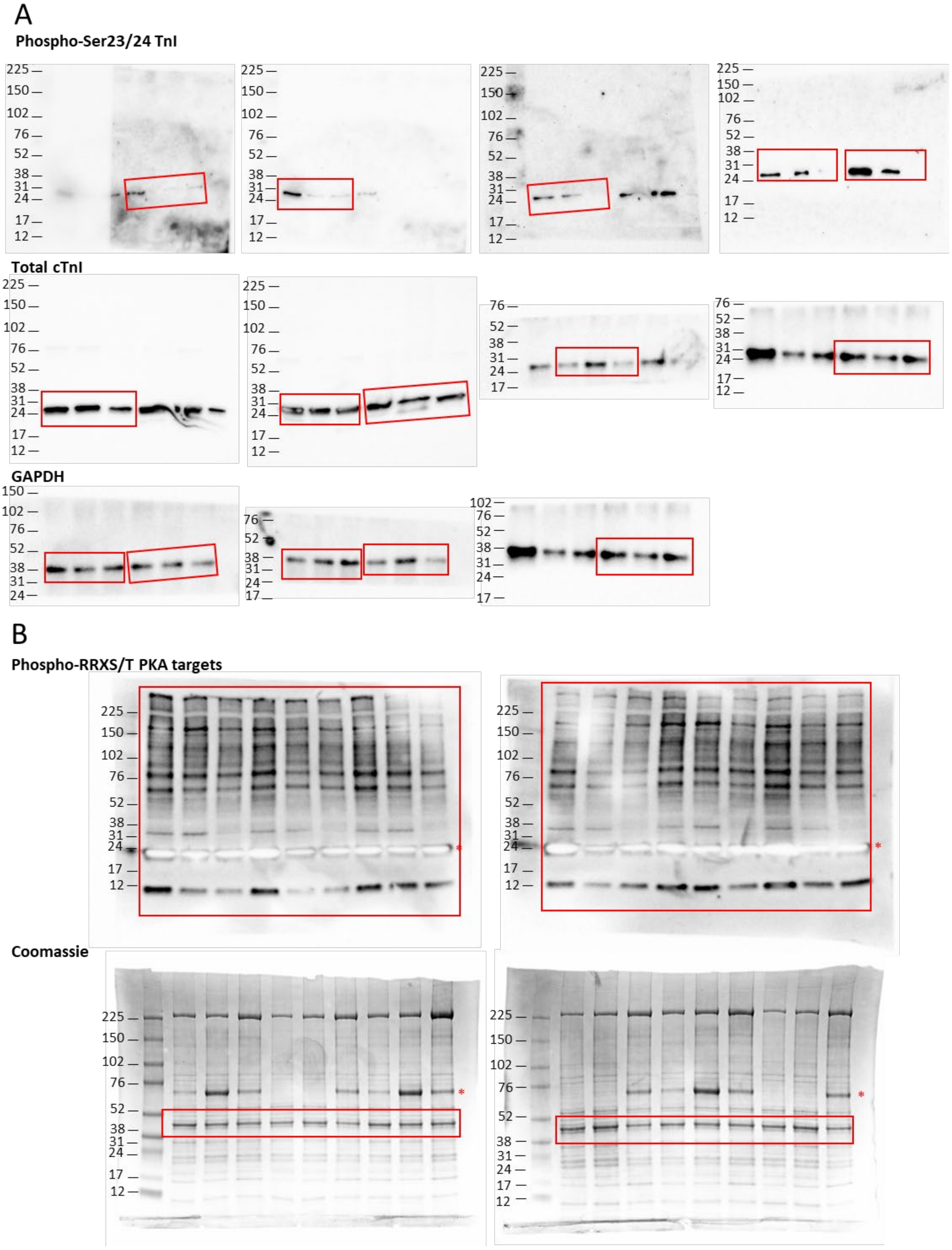

References

1 Gehmlich, K. *et al.* Changes in the cardiac metabolome caused by perhexiline treatment in a mouse model of hypertrophic cardiomyopathy. *Mol Biosyst* **11**, 564-573, doi:10.1039/c4mb00594e (2015).

2 Toepfer, C. N. *et al.* Hypertrophic cardiomyopathy mutations in MYBPC3 dysregulate myosin. *Sci Transl Med* **11**, doi:10.1126/scitranslmed.aat1199 (2019).

3 Hooijman, P., Stewart, M. A. & Cooke, R. A new state of cardiac myosin with very slow ATP turnover: a potential cardioprotective mechanism in the heart. *Biophys J* **100**, 1969-1976, doi:10.1016/j.bpj.2011.02.061 (2011).

4 Abou Tayoun, A. N. *et al.* Recommendations for interpreting the loss of function PVS1 ACMG/AMP variant criterion. *Hum Mutat* **39**, 1517-1524, doi:10.1002/humu.23626 (2018).

5 Richards, S. *et al.* Standards and guidelines for the interpretation of sequence variants: a joint consensus recommendation of the American College of Medical Genetics and Genomics and the Association for Molecular Pathology. *Genet Med* **17**, 405-424, doi:10.1038/gim.2015.30 (2015).
